# The impact of SGLT2 inhibitors on cardiac remodeling after myocardial infarction: an updated meta-analysis of randomized controlled trials

**DOI:** 10.3389/fphar.2025.1699066

**Published:** 2025-11-27

**Authors:** Ma Li Xu, Hui Wang, Dan Ouyang, Hang Qi, Xin Hui Li

**Affiliations:** 1 College of Traditional Chinese Medicine, Hunan University of Chinese Medicine, Changsha, China; 2 The Second Affiliated Hospital, Hunan University of Chinese Medicine, Changsha, China

**Keywords:** acute myocardial infarction, SGLT2 inhibitors, sodium-glucose-linked transporter 2 inhibitors, SGLT2i, meta-analysis

## Abstract

**Background:**

Recent studies show that sodium-glucose linked transporter 2 inhibitors (SGLT2is) reduce blood glucose and provide cardiovascular benefits, decreasing acute myocardial ischemia/reperfusion injury in patients with acute myocardial infarction (AMI).

**Objective:**

This meta-analysis aims to thoroughly assess the clinical effectiveness of SGLT2is in the treatment of AMI.

**Methods:**

Randomized controlled trials (RCTs) evaluating the efficacy of SGLT2is in combination with guideline-directed medical therapy (GDMT) for AMI were retrieved from major databases: PubMed, Cochrane Library, Embase, Medline, and Web of Science. At the same time, clinical trial registries (ClinicalTrials.gov and the WHO International Clinical Trials Registry Platform) were searched, covering all published literature up to May 2025. Using the Cochrane Collaboration for assessing the risk of bias, two independent reviewers preliminarily screened and assessed the studies according to the preset inclusion criteria. Meta-analysis was conducted using RevMan 5.4 software, and StataMP 16.0 was used to evaluate publication bias. The quality of evidence was graded according to recommended procedures for assessing and evaluating the evidence.

**Results:**

Five RCTs with a total of 881 patients were included in this analysis. According to a meta-analysis, SGLT2is and GDMT significantly reduced NT-proBNP (RR = −89.82, 95% CI -96.28 to −83.35; p < 0.00001) and enhanced the 12-week left ventricular ejection fraction (LVEF) (RR = 6.32, 95% CI -4.95 to 17.60; p < 0.00001). Evaluation of additional cardiac structural and functional characteristics showed that the SGLT2i + GDMT group showed significantly reduced left atrial volume (LAV) (RR = −3.86, 95% CI -6.33 to −1.38; p = 0.002) and left atrial volume index (LAVI) (RR = −1.67, 95% CI -3.13 to −0.20; p = 0.03) when compared to the control group. There were decreases in LVESVI, LVEDVI, LVEDD, LVESD, LVESV, and LVEDV. Furthermore, subgroup analyses based on the LVEF at admission and the site of the infarct in AMI patients were carried out. Treatment with SGLT2i + GDMT led to a significantly higher improvement in the LVEF ≤40% group than in the LVEF >40% group (MD = 5.20, 95% CI 2.74 to 7.66; p < 0.0001). The cardiotonic troponin I (cTnI) levels in the LVEF >40% group showed a declining trend starting at 8 h post-onset and a notable improvement at 40 h post-onset. Significant improvement in cTnI levels was observed in the LVEF ≤40% group after 56 h post-onset (MD = −8.40, 95% CI −13.74 to −3.06; p = 0.002). Regarding the effect of the infarct location on LVEF recovery, patients with AMI treated with SGLT2i + GDMT demonstrated a significant improvement in LVEF, regardless of whether the myocardial infarction was in the anterior wall (MD = 4.20, 95% CI 0.88 to 7.52; p = 0.01) or the non-anterior wall (MD = 3.90, 95% CI 0.63 to 7.17; p = 0.02). As early as 16 h after commencement, both groups’ cTnI levels showed a declining trend. By 24 h after the onset, non-anterior myocardial infarction patients showed a substantial improvement in cTnI levels (MD = −1.70, 95% CI −11.92 to −2.28; p = 0.004). However, ST-segment resolution showed no significant differences between the two groups. The SGLT2i + GDMT group’s incidence rate for the primary endpoint major adverse cardiovascular events (MACEs) was nearly identical to that of the control group. Recurrent myocardial infarction (RR = 0.64, 95% CI 0.16–2.55; p = 0.53), stroke (RR = 2.71, 95% CI 0.11–68.25; p = 0.54), and cardiovascular disease-related death (RR = 1.47, 95% CI 0.29–7.56; p = 0.64) did not differ significantly from one another. The incidence of MACEs in the experimental group was essentially comparable to that in the control group. For other primary endpoints, the incidence of re-admission for heart failure showed a downward trend in the experimental group compared with the control group. Furthermore, although no significant hepatic or renal dysfunction was reported in the studies, meta-analysis indicated that SGLT2i combined with GDMT increased the incidence of drug-related adverse events, which primarily manifested as higher rates of genitourinary infections and acute kidney injury (RR = 1.88, 95% CI 1.03–3.42; p = 0.04).

**Conclusion:**

Available data suggest that SGLT2i intervention may ameliorate detrimental early ventricular remodeling in individuals who have had an AMI, improve cardiac function, and aid in the recovery of cardiac function and structure.

## 1 Introduction

Acute myocardial infarction (AMI) can lead to adverse ventricular remodeling, subsequently triggering heart failure (HF). AMI is a major cause of HF worldwide, accounting for a significant proportion of cardiovascular disease-related deaths. Pathological cardiac remodeling constitutes an irreversible adaptive response triggered by multiple pathological states, including myocardial infarction (MI), ischemia/reperfusion (I/R) injury, pressure overload, inflammation, and oxidative stress. Its immediate consequences encompass myocardial hypertrophy and cardiac fibrosis, while prolonged adverse remodeling may ultimately result in HF. Progressive ventricular dys-remodeling, characterized by ventricular dilatation and impaired contractility, heralds post-infarction HF. The onset of HF following AMI correlates with significant morbidity and mortality. Advances in patient prognosis have slowed down over the last 15 years, with emergence of limited novel therapeutic options.

**FIGURE 1:**
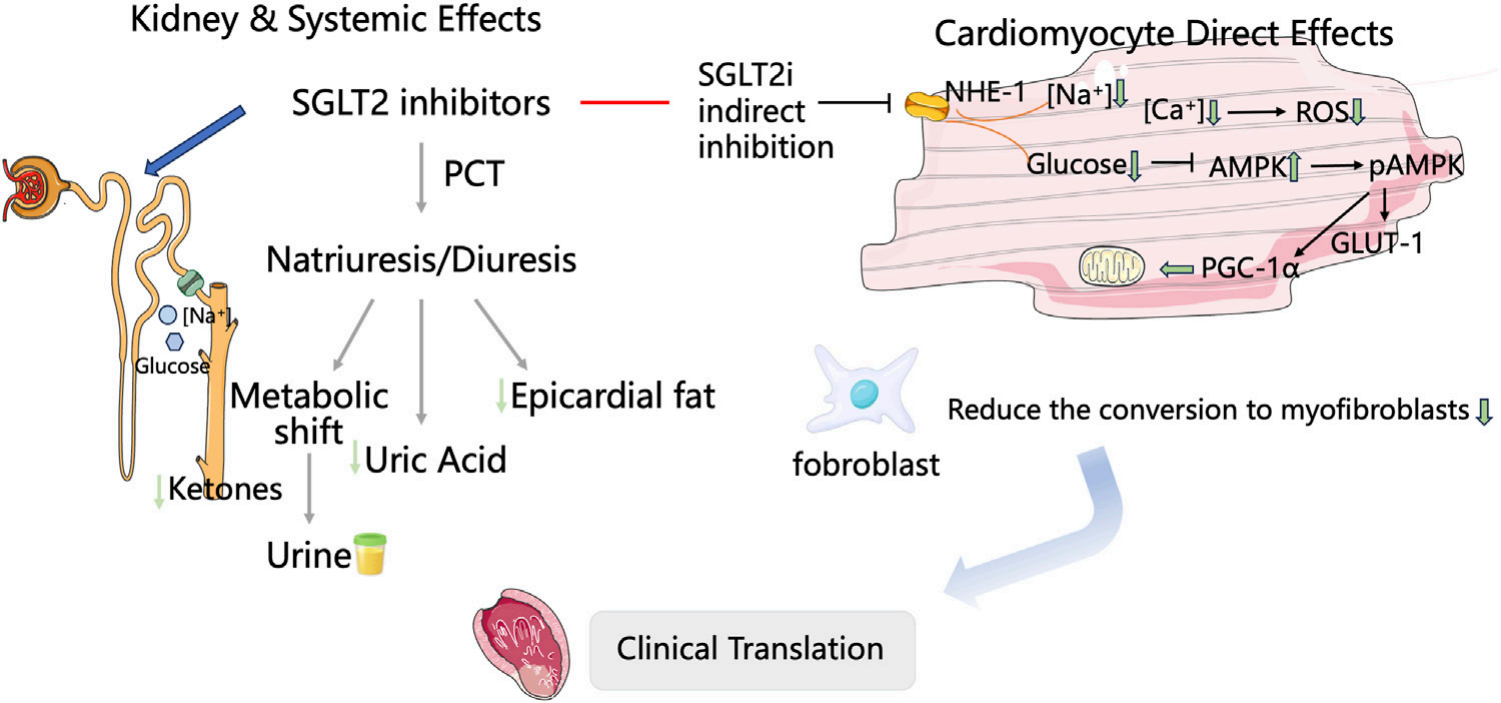
Proposed cardioprotective mechanisms of SGLT2 inhibitors following acute myocardial infarction.

Sodium-glucose linked transporter 2 inhibitors, SGLT2is (SGLT2 inhibitors) regulate the plasma glucose levels by inhibiting the reabsorption of glucose and sodium in the proximal renal tubules. Although initially used mainly for treating type 2 diabetes, numerous studies, including EMPA-REG OUTCOME, CANVAS-R, DECLARE, DAPA-HF, and EMPEROR-Reduced, have shown that SGLT2i significantly decreases the risk of cardiovascular event mortality (Kaplinsky, 2020; Mima, 2018; Perseghin and Solini, 2016; Verma et al., 2020). As indicated in the 2022 AHA/ACC/HFSA Heart Failure Management Guidelines (Heidenreich et al., 2022), SGLT2i is recommended for the treatment of symptomatic HF and patients with type 2 diabetes. It is the only medication that covers the entire management of HF, thereby achieving a comprehensive approach to the treatment of HF from a diabetes perspective. As SGLT2 is not expressed in cardiomyocytes, it was previously thought that SGLT2i could not directly act on cardiac muscle cells. Their effects were mainly attributed to systemic cardiovascular benefits achieved through osmotic diuresis, which reduces tissue fluid retention, decreases epicardial fat, and improves ketone body metabolism (Palmer and Clegg, 2021; Huang et al., 2023; van der Aart-van der Beek et al., 2022). Recent studies show that SGLT-2 inhibitors exert direct cardioprotective effects via multiple intracellular mechanisms. The inhibition of sodium/hydrogen exchanger-1 (NHE-1) lowers intracellular Na^+^ and Ca^2+^ levels, reduces CaMKII activity, enhances mitochondrial function, increases ATP production, and ultimately relieves HF (Mizushima and Komatsu, 2011). Additionally, increased phosphorylation of AMPK, PGC-1α, and STAT3 is linked to a decrease in reactive oxygen species (ROS) production and better endothelial-dependent vasorelaxation. Phosphorylation of myosin regulatory proteins helps improve myocardial relaxation, while activation of glucose transporter 1 (GLUT-1) supports cellular energy metabolism and vitality (Wichaiyo and Saengklub, 2022; Packer, 2022; Chen et al., 2022; Andrianesis and Doupis, 2013). Further studies suggest that SGLT-2 inhibitors may suppress or reverse cardiac remodeling through multiple mechanisms involving reduced cardiac inflammation, oxidative stress, endoplasmic reticulum stress, and apoptosis (Mooradian and Haas, 2022; Liu et al., 2021; Dai et al., 2023). These findings broaden the therapeutic scope of SGLT-2 inhibitors in cardiovascular disease. Preclinical studies indicate that SGLT2 inhibitors can mitigate acute myocardial ischemia/reperfusion injury, reduce the infarct size, enhance left ventricular function, and decrease arrhythmias (Andreadou et al., 2020; Lahnwong et al., 2020). In 2022, a multicenter, randomized, double-blind, placebo-controlled trial (EMMY) evaluated the efficacy and safety of empagliflozin in patients with AMI. The study demonstrated that empagliflozin significantly reduced NT-proBNP levels (−15%), improved the left ventricular ejection fraction (LVEF) by +1.5%, and improved structural parameters (reduction in LVESV/LVEDV ratio), with a favorable safety profile. In the multicenter, international observational cohort study (SGLT2-I AMI PROTECT, ClinicalTrials.gov identifier: NCT05261867), the use of SGLT2i was identified as an independent predictor of reduced major adverse cardiovascular events (MACEs) and an independent predictor of HF hospitalization rates (Paolisso et al., 2023). Nevertheless, comprehensive trials evaluating the efficacy and safety of SGLT2is in cardiac remodeling among AMI patients remain lacking. Only four meta-analyses have previously examined the role of SGLT2is in this context, though these studies (Usman et al.,2024; Hu et al., 2025; Li et al., 2024; Alizadehasl et al., 2025) possess certain limitations. These meta-analyses primarily focused on the impact of SGLT2i on hard endpoints related to HF, such as all-cause mortality and re-hospitalization, in patients diagnosed with AMI, providing insufficient primary outcome measures relevant to clinical prognosis. To address these limitations and fill gaps in the existing evidence, this meta-analysis aims to systematically evaluate the efficacy and safety of SGLT2is in treating cardiac remodeling in AMI patients (Figure 1).

## 2 Materials and methods

### 2.1 Protocol

**FIGURE 2:**
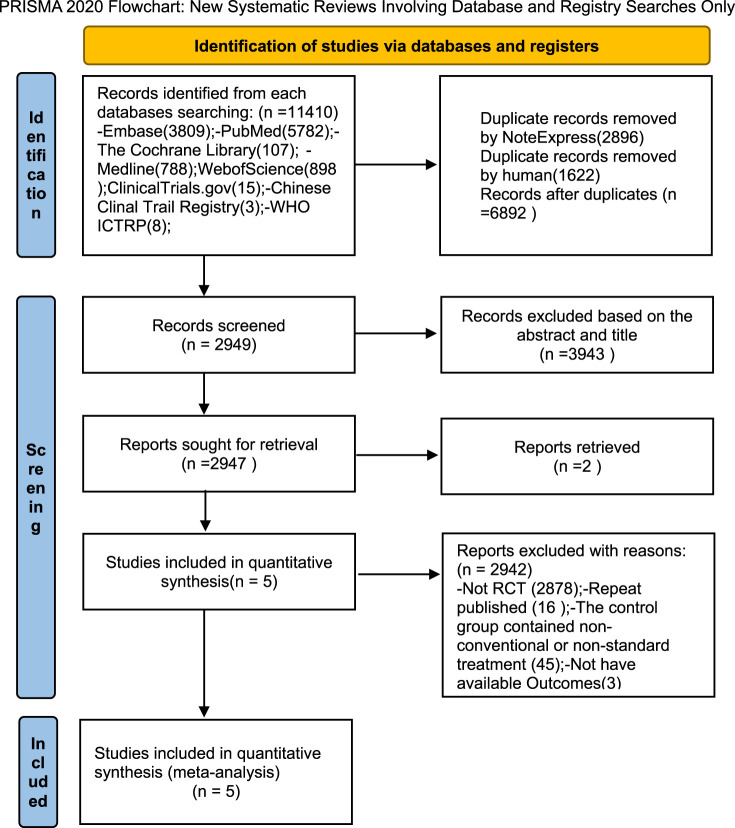
Flow diagram.

This meta-analysis was registered in the PROSPERO registry of systematic reviews (registration number CRD420251037822) on 23 April 2025 and conducted according to the Preferred Reporting Items for Systematic Reviews and Meta-Analyses (PRISMA) guidelines (Figure 2).

### 2.2 Protocol amendments

The following analyses represent enhancements or adaptations made to address the characteristics of the available data from the included randomized controlled trials (RCTs) and were undertaken to provide a more comprehensive and clinically relevant interpretation of the evidence.

#### 2.2.1 Expanded primary outcomes

The registered protocol specified the left ventricular end-systolic volume index (LVESVI) and the left ventricular end-diastolic volume index (LVEDVI) as the primary outcomes. In the final analysis, we reported a broader panel of key cardiac structural and functional parameters (including LVEF, NT-proBNP, LAV, and LAVI) as primary outcomes. This decision was made *post hoc* because these additional metrics are critically important for a holistic assessment of cardiac remodeling and were consistently reported across the included RCTs. The analysis of the prespecified primary outcomes (LVESVI and LVEDVI) is presented in Sections 3.4.3 and 3.4.4.

#### 2.2.2 *Ad hoc* subgroup analyses

The pre-planned subgroup analyses based on the MI type (STEMI vs. NSTEMI) and the intervention timing (≤7 days vs. >7 days post-MI) specified in the PROSPERO protocol could not be performed due to insufficient reporting of outcome data stratified by these variables in the primary studies. To robustly explore potential sources of heterogeneity and treatment effect modification, we conducted additional subgroup analyses based on baseline the LVEF and infarct location (anterior vs. non-anterior) because these patient-level factors were well-reported and are established determinants of post-infarct remodeling.

#### 2.2.3 Extended literature search

The search strategy was rigorously applied as planned. Furthermore, the Web of Science database was added to the search to enhance the comprehensiveness of the study identification process.

These amendments were undertaken to ensure that the review fully and accurately synthesized the current evidence landscape. They did not alter the fundamental objective of the review and provided supplementary insights that strengthen the clinical applicability of our findings.

### 2.3 Literature search strategy

The following databases were searched from their inception to May 2025: PubMed, Cochrane Library, Embase, Medline, Web of Science, and two clinical trial registries: ClinicalTrials.gov and the WHO Clinical Trials Registry Platform. No restrictions were imposed on the publication country or language. Additionally, relevant articles were retrieved from the reference lists of systematic reviews and included studies. The search strategy is detailed in Supplementary Table S1.

### 2.4 Selection criteria

#### 2.4.1 Inclusion criteria

All RCTs evaluating the efficacy and safety of SGLT2is, either as monotherapy or in combination with guideline-directed medical therapy, for the treatment of patients with AMI were included.

1. Study population: 1. Adults aged 18 years or older diagnosed with AMI, including ST-segment elevation MI (STEMI) and non-ST-segment elevation MI (NSTEMI), based on the fourth edition of the Universal Definition of Myocardial Infarction. 2. Patients with AMI within ≤14 days from symptom onset at intervention initiation. 3. Patients with or without comorbidities (e.g., type 2 diabetes, hypertension, and chronic kidney disease). 4. Revascularization methods: Patients with STEMI/NSTEMI undergoing PCI, thrombolysis, or medical therapy were included regardless of the revascularization status.
2. Intervention and control: The experimental group received any dose of SGLT2i (e.g., empagliflozin, dapagliflozin, or canagliflozin) orally following MI. The control group received standard of care (SOC) management following MI (without SGLT2is), including but not limited to the following: 1. antiplatelet therapy (e.g., aspirin and P2Y12 inhibitors). 2. Statins for lipid management. 3. Angiotensin-converting enzyme inhibitors (ACEIs)/angiotensin receptor blockers (ARBs) or angiotensin receptor-neprilysin inhibitors (ARNIs) for the renin–angiotensin–aldosterone system (RAAS) suppression. 4. Beta-blockers for heart rate and blood pressure control. Concomitant medication restrictions: Standard post-MI medications must be consistent across all groups.
3. Outcome measures: The primary outcomes included the left atrial volume (LAV), left atrial volume index (LAVI), left ventricular mass (LVM), left ventricular mass index (LVMI), left ventricular end-diastolic diameter (LVEDD), left ventricular end-systolic diameter (LVESD), left ventricular end-systolic volume), LVESVI, left ventricular end-diastolic volume (LVEDV), LVEDVI, LVEF, ePASP (estimated pulmonary artery systolic pressure), LV mass index (left ventricular mass index), and N-terminal pro-brain natriuretic peptide (NT-proBNP). The secondary outcomes included the infarct size, high-sensitivity troponin I (hs-TnI), and high-sensitivity C-reactive protein (hs-CRP). The primary endpoint was a composite of major adverse cardiovascular and cerebrovascular events (MACE), which is defined as follows: cardiovascular disease-related death, recurrent MI, and stroke. The secondary endpoints included readmission for HF and adverse drug reactions.

#### 2.4.2 Exclusion criteria

In addition to articles that did not meet the established criteria for inclusion, the following types of documents were excluded: observational studies (cohort studies); case reports, case series, editorials, letters to the editor, reviews, and non-controlled studies (e.g., single-arm trials); studies without full-text availability or in non-English languages (unless translation is feasible); and preclinical studies (animal models) or *in vitro* studies.

1. Subject exclusions: 1. Patients with pre-existing HF (NYHA class ≥ II) or cardiomyopathy, unless the study specifically addresses HF progression following MI. 2. Patients with type 1 diabetes mellitus. 3. Participants with severe renal impairment at baseline (estimated glomerular filtration rate [eGFR] <30 mL/min/1.73 m^2^), unless the study explicitly included this subgroup. 4. Participants with proven allergies or intolerance to SGLT2is, among other contraindications. 5. Nursing or pregnant women. 6. Active severe infection, uncontrolled hypertension (systolic blood pressure ≥180 mmHg), or malignancy affecting cardiac function. 7. Individuals unable to undergo cardiac imaging (e.g., contraindications to contrast agents for CMR/echocardiography). 8. Participants with a history of severe valvular heart disease (e.g., severe aortic stenosis/regurgitation) or congenital heart disease affecting the assessment of left ventricular remodeling. 9. Patients with a history of cardiac surgery (e.g., coronary artery bypass grafting or valve replacement), unless the study specifically targets post-MI populations.
2. Exclusion of interventions and controls: 1. Studies combining SGLT2is with other investigational drugs known to influence cardiac remodeling (e.g., novel anti-fibrotic agents and gene therapies), unless data for SGLT2is used alone can be independently extracted. 2. Non-oral formulations of SGLT2is (e.g., intravenous or topical) because oral administration constitutes the standard route for these agents. 3. Studies employing SGLT2is within multidrug combination interventions lacking a clear control group to assess the effect of SGLT2is alone. 4. Trials with undefined or inconsistent SGLT2i dosing (e.g., daily doses that were not fixed or standardized). 5. Preclinical studies (animal models) or *in vitro* research (excluding human clinical trials). 6. Studies evaluating SGLT2is in combination with hypoglycemic agents that may interfere with metabolic outcomes (e.g., GLP-1 receptor agonists), unless subgroup data for SGLT2i monotherapy are available. 7. Interventions involving SGLT2is where ≥20% of participants discontinued early due to adverse events, unless the primary analysis includes intention-to-treat data. 8. Placebo groups receiving investigational drugs directly affecting cardiac remodeling (e.g., novel anti-fibrotic agents), unless explicitly separated in subgroup analyses.

### 2.5 Data extraction and quality assessment

References identified through literature searches were managed using EndNote X9 software. Two authors independently reviewed the titles and abstracts of all papers retrieved from the specified databases to assess their potential eligibility. Publications that were duplicates or did not meet the inclusion criteria, including those related to the study’s interventions or outcomes, were excluded.

Following the screening of eligible studies, two researchers manually extracted data from the selected papers. Disagreements arising during data extraction were resolved through discussion, with a third author providing arbitration where necessary. Information collected encompassed the following categories: first author, year of publication, sample size, diagnostic criteria, participant characteristics (age and sex), trial design, intervention *versus* control groups, study methods, primary outcome measures, and reported adverse events. The methodological quality of the included studies was assessed using the Cochrane Collaboration’s risk of bias assessment tool. The assessment focused on multiple key domains, including random sequence generation, allocation concealment, blinding of participants and personnel, blinding of outcome assessors, management of incomplete outcome data, selective reporting, and other potential sources of bias. Each domain was categorized as “low risk,” “high risk,” or “unclear risk” based on the information provided by the included studies.

### 2.6 Statistical analysis

Statistical analysis was conducted using RevMan 5.4 and StataMP 16.0 software. Heterogeneity testing was performed initially, employing the I^2^ statistic and chi-square test to assess the significance and heterogeneity. Where heterogeneity testing yielded non-statistically significant results (p > 0.1, I^2^ < 50%), a fixed-effects model was applied; otherwise, a random-effects model was used. Subgroup analyses were conducted based on differences in control group interventions to identify potential sources of heterogeneity. Binary variables were expressed as odds ratios (ORs) or risk ratios (RRs), while continuous variables were presented as mean differences (MDs) or standardized mean differences (SMDs). All effect sizes included the corresponding 95% confidence intervals (95% CI). The significance threshold for meta-analysis was set at p ≤ 0.05 for the primary outcomes. Publication bias was assessed using Egger’s test, Harbord’s test, or trim-and-fill analysis; no publication bias was detected when p > 0.1. Sensitivity analyses examined changes in RR (OR) and MD (SMD) across all assessed outcomes following modifications to the employed effect model. Evidence quality was assessed using the GRADE tool (GRADEpro, 2015) according to the Grading of Recommendations, Assessment, Development and Evaluation (GRADE) manual.

## 3 Results

### 3.1 Literature screening results

Using established literature retrieval methods, 11,384 relevant studies were initially identified from databases. An additional 26 records were discovered from other sources (the China Clinical Trials Registry Platform, ClinicalTrials.gov, and the World Health Organization International Clinical Trials Registry Platform (WHO ICTRP)). Following the removal of 2,896 duplicate records via NoteExpress and the manual elimination of a further 1,622 duplicates, a total of 6,892 papers were retained for subsequent screening. Title and abstract screening resulted in the exclusion of 3,943 papers: 2,854 were non-RCTs and 1,089 involved inappropriate interventions. Two reports were identified as not retrieved. Five full-text articles were selected for comprehensive assessment. A further 2,942 papers were excluded for the following reasons: 2,878 were non-RCTs, 16 were duplicate publications, 45 included non-conventional or non-standard treatments in the control group, and three employed inappropriate outcome measures. Ultimately, five full-text articles were included in the final analysis (Figure 2).

### 3.2 Basic characteristics of the included studies

The five included studies encompassed 881 participants, and their basic characteristics are summarized in Table 1. Among these, 441 patients were assigned to the SGLT2i + guideline-directed medical therapy (GDMT) group, while 440 patients were assigned to the GDMT group. All included trials were registered at Tabriz University of Medical Sciences, Iran: EMI STEMI. (Khani et al., 2024). Iranian Registry of Clinical Trials Platform (IRCT20111206008307N42); Faculty of Medicine, Ain Shams University, Cairo, Egypt (DACAMI. Dayem et al., 2023; ClinicalTrials.gov [NCT05424315]); NHS Golden Jubilee National Hospital, UK (EMPRESS-MI, Carberry et al., 2025; ClinicalTrials.gov [NCT05020704]); and the EMMY trail, conducted across 11 research centers in Austria, led by Medical University of Graz, Austria: EMMY von Lewinski et al., (2022) ClinicalTrials.gov (NCT03087773); and China’s Shibei Hospital of Jingan District, Shanghai, by Zhou et al. (2024). Sample sizes ranged from 100 to 476 participants. However, notably, most studies had relatively small sample sizes. The participants’ mean ages ranged from 18 to 80 years. Five distinct diagnostic criteria for AMI were employed across the studies: three trials (Zhou et al., 2024; Carberry et al., 2025; Dayem et al., 2023) adhered to the Fourth Universal Definition of Myocardial Infarction (2018) (Thygesen et al., 2018). The remaining trials did not explicitly specify their AMI diagnostic criteria. All studies employed a two-group design: an intervention group and a GDMT group. In the intervention group, patients received SGLT2is combined with GDMT. The control group received standard therapy alone, comprising antiplatelet therapy, anti-ischemic drugs, and statins.

**TABLE 1:** Basic characteristics of the included literature.

Source	Randomly assigned	Allocation concealment	Double-blind	Single-blind	Cases of loss	Selective expression	Sample size		Intervention and dose			
							Trail group	Control group				
Zhou et al., 2024	Refer to random	Not described	Not mention	Not mentioned	Low risk	Low risk	53 (29/24)	47 (28/19)	10 mg dapagliflozin qd. po vs. CT			
von Lewinski et al., 2022	Refer to random	Not described	Double-blind	-	12	Low risk	237(195/42)	239(197/42)	10 mg empagliflozin, qd, po vs. CT			
Carberry et al., 2025	Refer to random	Not described	Double-blind	-	5	Low risk	51 (44/7)	53 (46/7)	10 mg empagliflozin, qd, po vs. CT			
Dayem et al., 2023	Refer to random	Not described	Double-blind	-	Low risk	Low risk	50(42/8)	50(41/9)	10 mg dapagliflozin, qd, po vs. CT			
Khani et al., 2024	Refer to random	Not described	Double-blind	-	5	Low risk	50(39/11)	51/40/11)	10 mg empagliflozin, qd, po vs. CT			

Main outcomes	Age (y)	Treatment duration	Disease state	Diagnostic criteria	Trial Registration	Types of myocardial infraction	Killip Classification		Coronary vessel angiography status		Infarct location. n(N)	
							Trail group	Control group	Trail group	Control group	Trail group	Control group
NT-pro BNP, LVEF, LVEDD, LVESD, adverse drug	>18	. 2 weeks	within 2 hours	-	-	-	Class (12) Class 11(26) Class 11(15)	Class (10) Class II(17) Class 11(19)	-	-	-	-
NT-proBNP	18–80	over 26 weeks	Within 72 h of percutaneous 5 coronary intervention	-	ClinicalTrials.gov (NCT03087 773)	STEMI& NSTEMI	-	-	3 vessel disease 50(2) 1); 2 vessel (disease 62(3) 4.6); 1 I disease 105(44.3)	3 vessel disease 36(1 5.0): 2 vessel disease 60(3 3.5): 1 vessel disease 123 51.5)	-	-
LVESV, left ventricular and atrial volumes, left ventricular mass, LVEF hs-Tnl NT-proBNP	63 ± 11	24 weeks	≥12 h and ≤14 days	Recommendations from the fourth universal definition of MI	ClinicalTrials.gov (NCT05020 704)	STEMI & NSTEMI	-	-	-	-	anterior:41(80.4);Inferior:6(11.8);Laterial:4(7.8)	anterior:42(79.2);Inferior:9(17.0);Laterial:2(3.8)
NT-pro BNP.LVEF. LVEDD LVESD. adverse drug reaction, MACE (cardiovascular death, recurrent myocardial infarction, and stroke)	55.24 ± 13.2	12 weeks	Within 72h of percutaneous and coronary intervention	Recommendations from the fourth universal definition of MI	ClinicalTrials.gov NCT05424315)	STEMI	Class (49)Class (1)Class II(0)	Class 1(48)Class (2)Class II(0)	-	-	proximal third:42;mid third:8	proximal third:39;mid third:11
cTnI, hs-CRP.LVEF. MACE reaction MACE (cardiovascular death, recurrent myocardial infraction, and stroke)	>18	40 days	within 90 min	-	Transan Registry of Clinical Trials Platform (IR) CT2011120 5008307N42)	STEMI	-	-	-	-	LAD:33.0,R CA:12:LCx1 Other 4	LAD:30.0,R CA:13:LCo4: Other. 4

Five studies reported the primary outcome LVEF (Zhou et al., 2024; von Lewinski et al., 2022; Carberry et al., 2025; Dayem et al., 2023; Khani et al., 2024). Four studies reported the primary outcome NT-proBNP (Zhou et al., 2024; von Lewinski et al., 2022; Carberry et al., 2025; Dayem et al., 2023). Three studies reported the secondary outcome LVEDD (Zhou et al., 2024; von Lewinski et al., 2022; Dayem et al., 2023). Two studies reported the primary outcome LAVI and the secondary outcome LAV (von Lewinski et al., 2022; Carberry et al., 2025). Two studies reported the secondary outcome LVESD (Zhou et al., 2024; Dayem et al., 2023). Two studies reported the primary outcomes LVESVI and LVEDVI and the secondary outcomes LVESV and LVEDV (von Lewinski et al., 2022; Carberry et al., 2025). One study reported the secondary outcome LVM and LVMI (Carberry et al., 2025). One study reported the secondary outcomes ePASP and LV mass index (Dayem et al., 2023). One study reported the secondary outcomes infarct size and hs-TnI (Carberry et al., 2025). One study reported the secondary outcome hs-CRP (Khani et al., 2024). Three studies reported the primary endpoint MACE (Zhou et al., 2024; von Lewinski et al., 2022; Khani et al., 2024). HF readmission was reported in four studies (Zhou et al., 2024; von Lewinski et al., 2022; Carberry et al., 2025; Dayem et al., 2023). Adverse drug reactions were reported in four studies (Zhou et al., 2024; von Lewinski et al., 2022; Carberry et al., 2025; Khani et al., 2024).

### 3.3 Risk-of-bias assessment

#### 3.3.1 Random sequence generation and allocation concealment

All included studies reported random allocation. Two studies (Khani et al., 2024; Carberry et al., 2025) employed permuted block randomization, while two studies (Dayem et al., 2023; von Lewinski et al., 2022) employed computer-generated randomization. These studies were categorized as having low risk of bias. For other RCTs that did not mention allocation concealment, the risk of bias was assessed as unclear.

#### 3.3.2 Blinding

Four studies (von Lewinski et al., 2022; Carberry et al., 2025; Dayem et al., 2023; Khani et al., 2024) employed double-blind designs and were, therefore, categorized as having a low risk of bias. In contrast, other RCTs did not mention blinding, suggesting potential selection bias. Furthermore, blinding in outcome assessment across all trials was not considered to influence the outcome evaluation, indicating an extremely low likelihood of detection bias.

#### 3.3.3 Incomplete outcome data and selective outcome reporting

Khani et al., 2024, Carberry et al., 2025; von Lewinski et al., 2022 exhibited incomplete outcome data, and reasons for missing data were provided. The number of missing outcome data points was balanced across the intervention groups, and the reasons for missing data were similar across groups; thus, we assessed the risk of bias as low. No study demonstrated selective outcome reporting; all RCTs were rated as having a low risk of bias.

#### 3.3.4 Other potential bias

**FIGURE 3:**
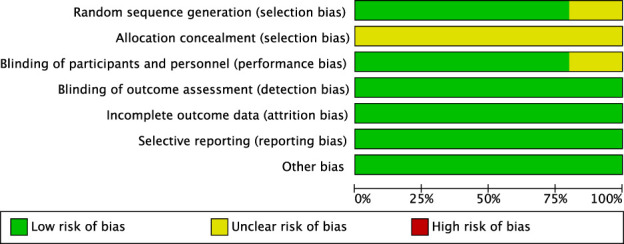
Risk of bias graph.

**FIGURE 4:**
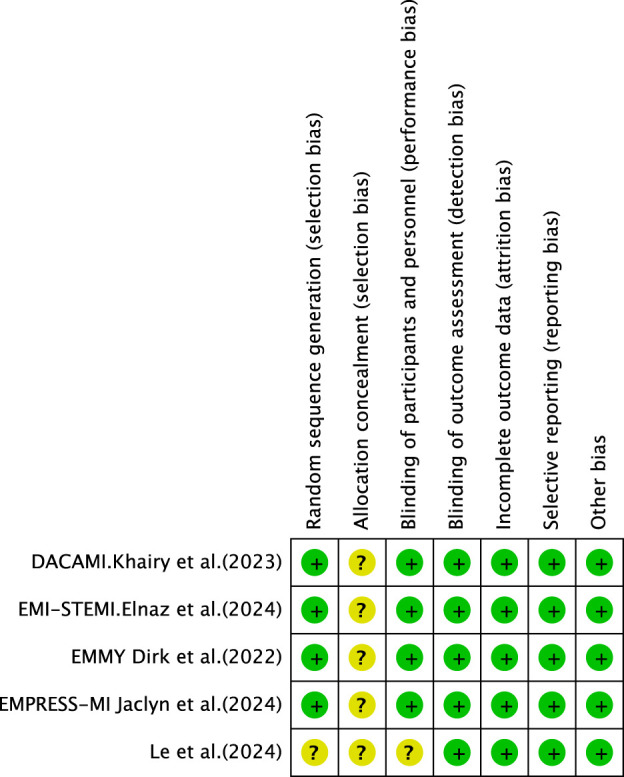
Risk of bias summary.

These RCTs were not affected by other sources of bias; consequently, we assessed their risk of bias as low. Further details are presented in Figures 3, 4.

### 3.4 Primary outcome measures

#### 3.4.1 LVEF

**FIGURE 5:**
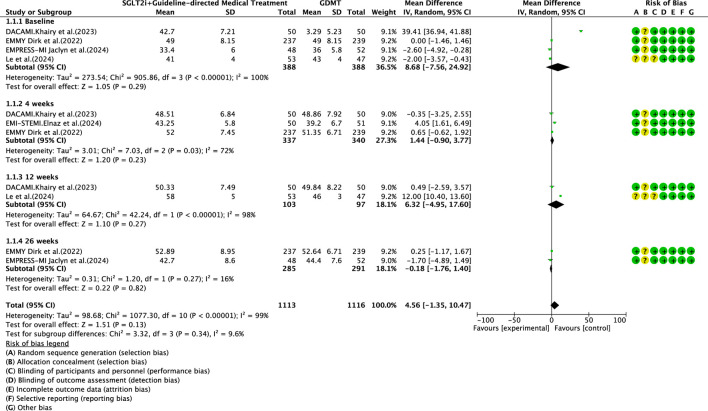
The outcomes of LVEF of SGLT2i + GDMT vs. GDMT.

Five studies (Zhou et al., 2024; von Lewinski et al., 2022; Carberry et al., 2025; Dayem et al., 2023; Khani et al., 2024) reported the primary outcome LVEF, involving 881 patients. Of these, 442 patients comprised the intervention group receiving SGLT2i in conjunction with GDMT, while 439 patients formed the control group receiving GDMT alone. The I^2^ test yielded χ^2^ = 1077.30, df = 10, p < 0.00001, and I^2^ = 99%, indicating substantial heterogeneity between the studies. Meta-analysis employed a random-effects model. The analysis revealed an upward trend in the experimental group compared to that in the control group, though the effect lacked statistical significance (RR = 4.56, 95% CI −1.35 to 10.47; p = 0.13). The five studies were categorized into four subgroups based on the treatment duration. Within the baseline subgroup, four studies (Zhou et al., 2024; von Lewinski et al., 2022; Carberry et al., 2025; Dayem et al., 2023) assessed baseline LVEF. The I^2^ test yielded χ^2^ = 905.86, df = 3, p < 0.00001, and I^2^ = 100%, indicating extremely high homogeneity and no statistical difference in baseline values between the groups. In the 4-week subgroup, three studies (von Lewinski et al., 2022; Carberry et al., 2025; Dayem et al., 2023) assessed LVEF at 4 weeks. The I^2^ test yielded χ^2^ = 7.03, df = 2, p = 0.03, and I^2^ = 72%, necessitating the use of a random-effects model. The results indicated a trend toward increased LVEF in the intervention group (RR = 1.44, 95% CI −0.9 to 3.77; p = 0.23). Within the 12-week subgroup, two trials (Zhou et al., 2024; Dayem et al., 2023) assessed LVEF at 12 weeks, yielding an I^2^ test result of χ^2^ = 42.24, df = 2, p = 0.03, and I^2^ = 72%, thus necessitating a random-effects model. The results indicated a significant increase in the intervention group compared to that in the control group (RR = 6.32, 95% CI −4.95 to 17.60; p < 0.00001). In the 26-week subgroup, two trials (von Lewinski et al., 2022; Carberry et al., 2025) assessed LVEF at 26 weeks. The I^2^ test yielded χ^2^ = 1.20, df = 1, p = 0.27, and I^2^ = 16%, using a random-effects model. The results indicated that LVEF in the experimental group was essentially comparable to that in the control group, with no statistically significant difference (RR = −0.18, 95% CI −1.76 to 1.40; p = 0.27), as shown in Figure 5.

#### 3.4.2 NT-proBNP

**FIGURE 6:**
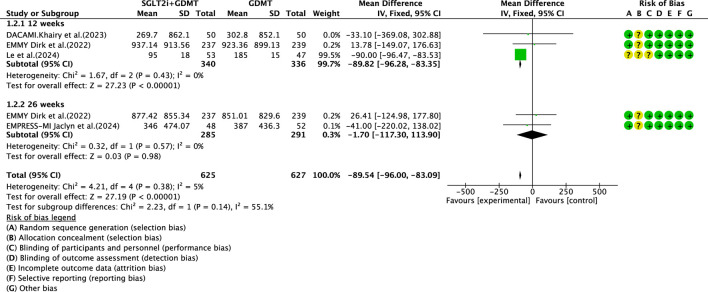
The outcomes of NT-proBNP of SGLT2i + GDMT vs. GDMT.

Four studies (Zhou et al., 2024; von Lewinski et al., 2022; Carberry et al., 2025; Dayem et al., 2023) reported the primary outcome NT-proBNP, involving 780 patients. Of these, 391 patients formed the experimental group receiving SGLT2i combined with GDMT, while 389 patients comprised the control group receiving GDMT alone. The I^2^ test yielded χ^2^ = 4.21, df = 4, p = 0.38, and I^2^ = 5%, indicating heterogeneity among studies. Meta-analysis employed a fixed-effects model. The analysis revealed a significant reduction in NT-proBNP levels in the experimental group compared to that in the control group (RR = −89.54, 95% CI -96.00 to −83.09; p < 0.00001). The four studies were subdivided into two subgroups based on the treatment duration. In the 12-week subgroup, three studies (Zhou et al., 2024; von Lewinski et al., 2022; Dayem et al., 2023) assessed NT-proBNP at 12 weeks. The I^2^ test yielded χ^2^ = 1.67, df = 2, p = 0.43, and I^2^ = 0%, necessitating the fixed-effects model. The results demonstrated a significant reduction in NT-proBNP in the intervention group (RR = −89.82, 95% CI -96.28 to −83.35; p < 0.00001). In the 26-week subgroup, two studies (von Lewinski et al., 2022; Carberry et al., 2025) assessed NT-proBNP at 26 weeks. The I^2^ test yielded χ^2^ = 0.32, df = 1, p = 0.57, and I^2^ = 0%, thus requiring a fixed-effects model. The results indicated a trend toward reduced NT-proBNP in the intervention group, though the difference was not statistically significant (RR = −1.70, 95% CI -117.30 to 113.90; p = 0.98), as shown in Figure 6.

#### 3.4.3 LVESVI

**FIGURE 7:**
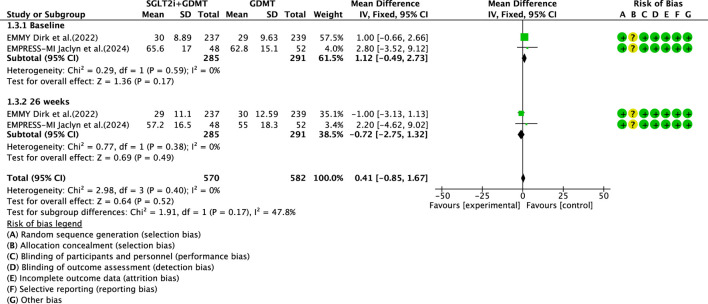
The outcomes of LVESVI of SGLT2i + GDMT vs. GDMT.

Two studies (von Lewinski et al., 2022; Carberry et al., 2025) reported the primary outcome LVESVI, involving 580 patients. Of these, 288 patients comprised the experimental group receiving SGLT2i combined with GDMT, while 292 patients formed the control group receiving GDMT alone. The I^2^ test yielded χ^2^ = 2.98, df = 3, p = 0.40, and I^2^ = 0%, indicating substantial heterogeneity between the studies. A fixed-effects meta-analysis was conducted. The results showed a trend toward reduced LVESVI in the experimental group compared to that in the control group (RR = 0.41, 95% CI −0.85 to 1.67; p = 0.52). Two studies were subdivided into subgroups based on the treatment timing. In the baseline subgroup, two studies (von Lewinski et al., 2022; Carberry et al., 2025) assessed LVESVI at baseline. The I^2^ test yielded χ^2^ = 0.29, df = 1, p = 0.59, and I^2^ = 0%, necessitating a fixed-effects model. The results indicated no statistically significant difference in LVESVI between the intervention and control groups (RR = 1.12, 95% CI −0.49 to 2.73; p = 0.17). In the 26-week subgroup, two studies (von Lewinski et al., 2022; Carberry et al., 2025) assessed LVESVI at 26 weeks. The I^2^ test yielded χ^2^ = 0.77, df = 1, p = 0.38, and I^2^ = 0%, necessitating a fixed-effects model. The results indicated a trend toward reduced LVESVI in the intervention group, though the effect lacked statistical significance (RR = −0.72, 95% CI −0.85 to 1.32; p = 0.49), as illustrated in Figure 7.

#### 3.4.4 LVEDVI

**FIGURE 8:**
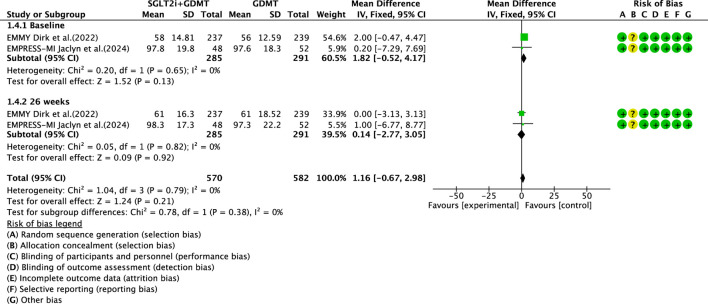
The outcomes of LVEDVI of SGLT2i + GDMT vs. GDMT.

Two studies (von Lewinski et al., 2022; Carberry et al., 2025) reported the primary outcome LVEDVI, involving 580 patients. Of these, 288 patients comprised the experimental group receiving SGLT2i combined with GDMT, while 292 patients formed the control group receiving GDMT alone. The I^2^ test yielded χ^2^ = 1.04, df = 3, p = 0.79, and I^2^ = 0%, enabling meta-analysis using a fixed-effects model. The results indicated no significant differences between the experimental and control groups (RR = 1.16, 95% CI −0.67 to 2.98; p = 0.21). Two studies were subdivided into subgroups based on the treatment duration. Within the baseline subgroup, two studies (von Lewinski et al., 2022; Carberry et al., 2025) assessed baseline LVEDVI. The I^2^ test yielded χ^2^ = 0.20, df = 1, p = 0.65, and I^2^ = 0%, necessitating a fixed-effects model. The results indicated comparable LVEDVI in the experimental group (RR = 1.81, 95% CI −0.52 to 4.17; p = 0.13). Within the 26-week subgroup, two studies (von Lewinski et al., 2022; Carberry et al., 2025) assessed LVEDVI at 26 weeks. The I^2^ test yielded χ^2^ = 0.05, df = 1, p = 0.82, and I^2^ = 0%, necessitating a fixed-effects model. The results indicated a trend toward reduced LVEDVI in the intervention group, though the effect lacked statistical significance (RR = 0.14, 95% CI −2.77 to 3.05; p = 0.92), as illustrated in Figure 8.

#### 3.4.5 LAVI

**FIGURE 9:**
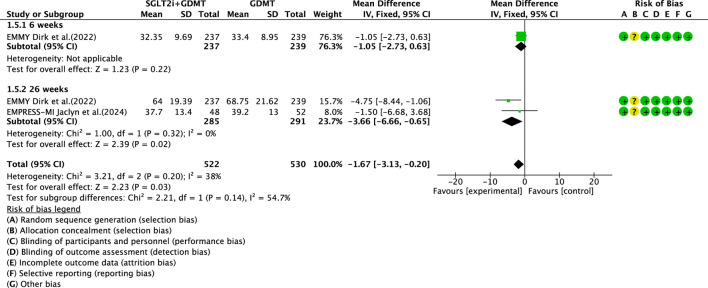
The outcomes of LAVI of SGLT2i + GDMT vs. GDMT.

Two studies (von Lewinski et al., 2022; Carberry et al., 2025) reported the secondary outcome LAVI, involving 580 patients. Of these, 288 patients comprised the experimental group receiving SGLT2i combined with GDMT, while 292 patients formed the control group receiving GDMT alone. The I^2^ test yielded χ^2^ = 3.21, df = 2, p = 0.20, and I^2^ = 38%. Meta-analysis employed a fixed-effects model. The results indicated a significant reduction in the intervention group compared to that in the control group (RR = −1.67, 95% CI −3.13 to −0.20; p = 0.03). Two studies were subdivided into subgroups based on the treatment duration. In the 6-week subgroup, only one study (von Lewinski et al., 2022; Carberry et al., 2025) assessed LAVI at 6 weeks, showing a trend toward reduced LAVI in the experimental group (RR = −1.05, 95% CI −2.73 to 0.63; p = 0.22). In the 26-week subgroup, both studies (von Lewinski et al., 2022; Carberry et al., 2025) assessed LAVI at 26 weeks. The I^2^ test yielded χ^2^ = 1.00, df = 1, p = 0.32, and I^2^ = 0%, necessitating a fixed-effects model. The results demonstrated a significant reduction in LAVI within the experimental group (RR = −3.66, 95% CI −6.66 to −0.65; p = 0.02), as illustrated in Figure 9.

### 3.5 Secondary outcome measures

#### 3.5.1 LVESD

**FIGURE 10:**
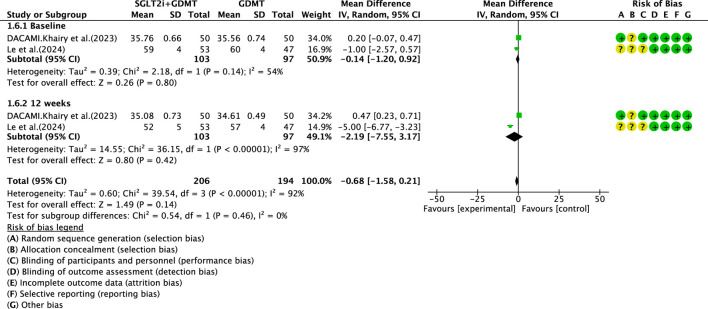
The outcomes of LVESD of SGLT2i + GDMT vs. GDMT.

Two studies (Zhou et al., 2024; Dayem et al., 2023) reported the secondary outcome LVESD, involving 200 patients. Of these, 103 patients comprised the experimental group receiving SGLT2i combined with GDMT, while 97 patients formed the control group receiving GDMT alone. The I^2^ test yielded χ^2^ = 39.54, df = 3, p < 0.00001, and I^2^ = 92%, necessitating meta-analysis using a random-effects model. The analysis revealed a trend toward reduction in the experimental group compared to that in the control group, though the effect lacked statistical significance (RR = −0.68, 95% CI −1.58 to 0.21; p = 0.14). The two studies were subdivided into two subgroups based on the treatment duration. In the baseline subgroup, two studies ((Zhou et al., 2024; Dayem et al., 2023) assessed baseline LVESD. The I^2^ test yielded χ^2^ = 2.18, df = 1, p = 0.14, and I^2^ = 54%, necessitating a random-effects model. The results indicated comparable LVESD between the groups (RR = −0.14, 95% CI −1.20 to 0.92; p = 0.80). In the 12-week subgroup, two studies ((Zhou et al., 2024; Dayem et al., 2023) assessed LVESD at 12 weeks. The I^2^ test yielded χ^2^ = 36.15, df = 1, p < 0.00001, and I^2^ = 97%, necessitating a random-effects model. The results indicated a trend toward reduced LVESD in the intervention group (RR = −2.19, 95% CI −7.55 to 3.17; p = 0.42), as depicted in Figure 10.

#### 3.5.2 LVEDD

**FIGURE 11:**
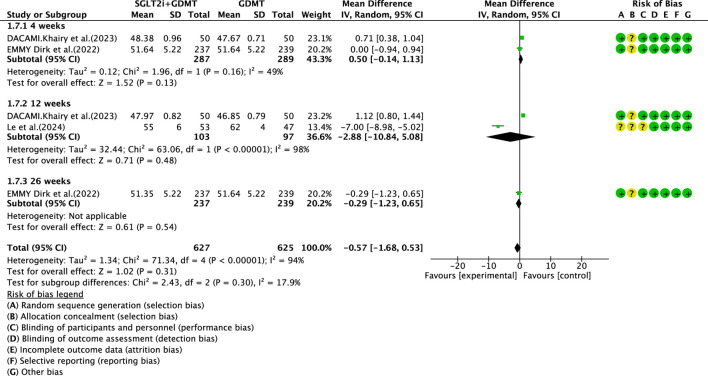
The outcomes of LVEDD of SGLT2i + GDMT vs. GDMT.

Three studies (Zhou et al., 2024; von Lewinski et al., 2022; Dayem et al., 2023) reported the secondary outcome LVEDD, involving 676 patients. Of these, 340 patients comprised the experimental group receiving SGLT2i combined with GDMT, while 336 patients formed the control group receiving GDMT alone. The I^2^ test yielded χ^2^ = 71.34, df = 4, p < 0.00001, and I^2^ = 94%. Meta-analysis employed a random-effects model. The analysis revealed a trend toward reduction in the experimental group compared to that in the control group (RR = −0.57, 95% CI −1.68 to 0.53; p = 0.31). The two studies were subdivided into two subgroups based on the treatment duration. In the 4-week subgroup, two studies (von Lewinski et al., 2022; Dayem et al., 2023) assessed LVEDD at 4 weeks, with the I^2^ test yielding χ^2^ = 1.96, df = 1, p = 0.16, and I^2^ = 49%. The results indicated a trend toward reduction in the intervention group compared to that in the control group (RR = 0.50, 95% CI −0.14 to 1.13; p = 0.13). In the 12-week subgroup, three studies (Zhou et al., 2024; von Lewinski et al., 2022; Dayem et al., 2023) assessed LVEDD at 12 weeks. The I^2^ test yielded χ^2^ = 63.06, df = 1, p < 0.00001, and I^2^ = 98%, necessitating the use of a random-effects model. The results indicated a trend toward reduced LVEDD in the intervention group (RR = −2.88, 95% CI −10.84 to 5.08; p = 0.48). Within the 26-week subgroup, only one study (von Lewinski et al., 2022) assessed LVEDD at 26 weeks. The results indicated a trend toward reduced LVEDD in the intervention group, though the effect lacked statistical significance (RR = −0.29, 95% CI −1.23 to 0.65; p = 0.54), as depicted in Figure 11.

#### 3.5.3 LVESV

**FIGURE 12:**
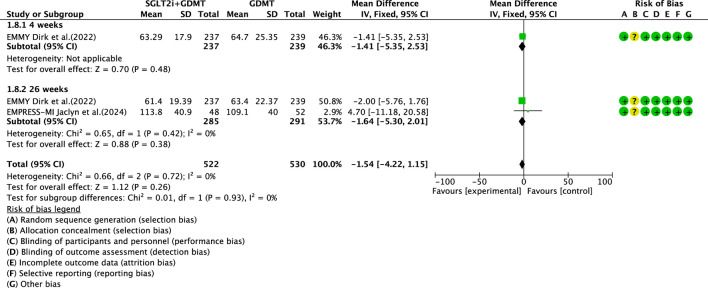
The outcomes of LVESV of SGLT2i + GDMT vs. GDMT.

Two studies (von Lewinski et al., 2022; Carberry et al., 2025) reported the secondary outcome LVESV, involving 580 patients. Of these, 288 patients comprised the experimental group receiving SGLT2i combined with GDMT, while 292 patients formed the control group receiving GDMT alone. The I^2^ test yielded χ^2^ = 0.66, df = 2, p = 0.72, and I^2^ = 0%. Meta-analysis employed a fixed-effects model. The results indicated a trend toward reduced LVESV in the experimental group compared to that in the control group (RR = −1.54, 95% CI −4.22 to 1.15; p = 0.26). The two studies were subdivided into two subgroups based on the treatment duration. In the 4-week subgroup, only one study (von Lewinski et al., 2022) assessed LVESV at 4 weeks, showing a trend toward reduced LVESV in the experimental group (RR = −1.41, 95% CI −5.35 to 2.53; p = 0.48). In the 26-week subgroup, both studies (von Lewinski et al., 2022; Carberry et al., 2025) assessed LVESV at 26 weeks. The I^2^ test yielded χ^2^ = 0.65, df = 1, p = 0.42, and I^2^ = 0%, necessitating the use of a fixed-effects model. The results indicated a trend toward reduced LVESV in the intervention group, though the effect lacked statistical significance (RR = −1.64, 95% CI −5.30 to 2.01; p = 0.38), as shown in Figure 12.

#### 3.5.4 LVEDV

**FIGURE 13:**
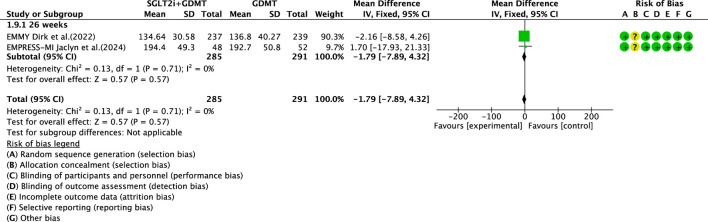
The outcomes of LVEDV of SGLT2i + GDMT vs. GDMT.

Two studies (von Lewinski et al., 2022; Carberry et al., 2025) reported the secondary outcome LVEDV, involving 580 patients. Of these, 288 patients comprised the experimental group receiving SGLT2i combined with GDMT, while 292 patients formed the control group receiving GDMT alone. In the 26-week cohort, the I^2^ test yielded χ^2^ = 0.13, df = 1, p = 0.71, and I^2^ = 0%. Meta-analysis employed a fixed-effects model. The results indicated a trend toward reduction in the experimental group compared to that in the control group (RR = −1.79, 95% CI −7.89 to 4.32; p = 0.57), as illustrated in Figure 13.

#### 3.5.5 LAV

**FIGURE 14:**
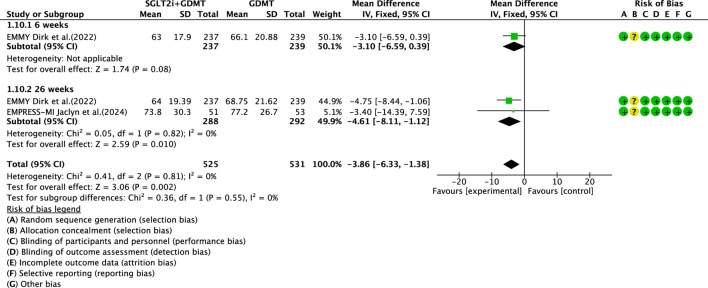
The outcomes of LAV of SGLT2i + GDMT vs. GDMT.

Two studies (von Lewinski et al., 2022; Carberry et al., 2025) reported the secondary outcome LAV, involving 580 patients. Of these, 288 patients comprised the experimental group receiving SGLT2i combined with GDMT, while 292 patients formed the control group receiving GDMT alone. The I^2^ test yielded χ^2^ = 0.41, df = 2, p = 0.81, and I^2^ = 0%. Meta-analysis employed a fixed-effects model. The results indicated a significant reduction in LAV in the experimental group compared to that in the control group (RR = −3.86, 95% CI −6.33 to −1.38; p = 0.002). The two studies were subdivided into two subgroups based on the treatment duration. In the 6-week subgroup, only one study (von Lewinski et al., 2022) assessed LAV at 6 weeks, showing a trend toward reduced LAV in the experimental group compared to that in the control group (RR = −3.10, 95% CI −6.59 to 0.39; p = 0.08). In the 26-week subgroup, both studies (von Lewinski et al., 2022; Carberry et al., 2025) assessed 26-week LAV. The I^2^ test yielded χ^2^ = 0.05, df = 1, p = 0.82, and I^2^ = 0%, necessitating the use of a fixed-effects model. The results demonstrated a significant reduction in LAV in the intervention group (RR = −4.61, 95% CI −8.11 to −1.12; p = 0.010), as illustrated in Figure 14.

#### 3.5.6 Other secondary outcome measures

Results for other secondary endpoints, including cardiac structural and functional parameters (LVM, LVMI, ePASP, LV mass index, MI markers (cardiotonic troponin I [cTnI]), inflammatory markers (high-sensitivity C-reactive protein [hs-CRP]), and MacNew HRQoL scores, are detailed in Supplementary Table S2.

### 3.6 Primary endpoint measures

#### 3.6.1 MACE

**FIGURE 15:**
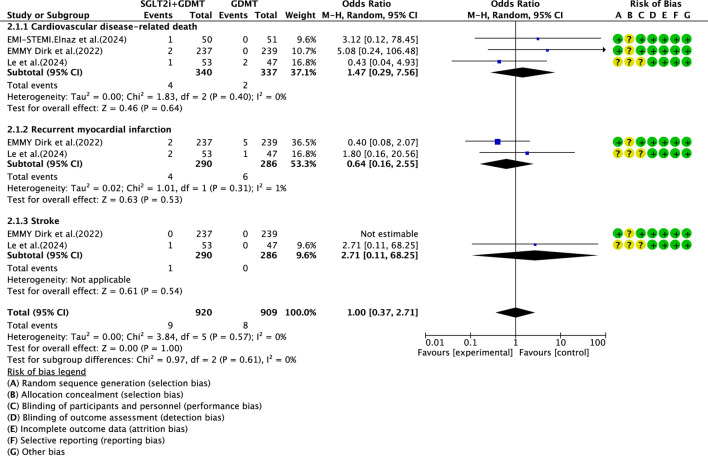
The outcomes of MACE of SGLT2i + GDMT vs. GDMT.

Three studies (Zhou et al., 2024; von Lewinski et al., 2022; Khani et al., 2024) reported the primary endpoint measure MACE, involving 677 patients. Among these, 340 patients comprised the experimental group receiving SGLT2i combined with GDMT, while 337 patients formed the control group receiving GDMT alone. The I^2^ test yielded χ^2^ = 3.84, df = 5, p = 0.57, and I^2^ = 0%. A fixed-effects model was employed for meta-analysis. The results indicated that the incidence rate in the experimental group was essentially comparable to that in the control group, with a statistically significant difference (RR = 1.00, 95% CI 0.37–2.71; p = 0.002). Three studies (Zhou et al., 2024; von Lewinski et al., 2022; Khani et al., 2024) reported cardiovascular disease-related death. Within this subgroup, the I^2^ test yielded χ^2^ = 0.05, df = 2, p = 0.40, and I^2^ = 0%, necessitating a fixed-effects model. The results indicated that the incidence of MACEs in the experimental group was essentially comparable to that in the control group (RR = 1.47, 95% CI 0.29–7.56; p = 0.64). Two studies (Zhou et al., 2024; von Lewinski et al., 2022) reported recurrent MI. The I^2^ test yielded χ^2^ = 0.05, df = 1, p = 0.82, and I^2^ = 0%, necessitating the use of a fixed-effects model. Within the recurrent MI subgroup, the I^2^ test yielded χ^2^ = 1.01, df = 1, p = 0.31, and I^2^ = 1%, thus necessitating a fixed-effects model. The results indicated comparable MACE incidence between the experimental and control groups (RR = 0.64, 95% CI 0.16–2.55; p = 0.53). Two studies (Zhou et al., 2024; von Lewinski et al., 2022) reported stroke outcomes. The results indicated no statistically significant difference in MACE incidence between the intervention and control groups (RR = 2.71, 95% CI 0.11–68.25; p = 0.54), as illustrated in Figure 15.

#### 3.6.2 Re-admission for heart failure

**FIGURE 16:**
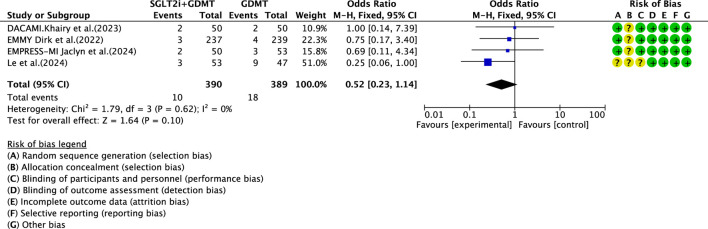
The outcomes of Re-admission for heart failure of SGLT2i + GDMT vs. GDMT.

Four studies (Zhou et al., 2024; von Lewinski et al., 2022; Carberry et al., 2025; Dayem et al., 2023) reported the primary endpoint measure of re-admission for HF, involving 779 patients. Of these, 390 patients formed the intervention group receiving SGLT2i combined with GDMT, while 389 patients comprised the control group receiving GDMT alone. The I^2^ test yielded χ^2^ = 1.79, df = 3, p = 0.62, and I^2^ = 0%, permitting meta-analysis using a fixed-effects model. The analysis revealed a downward trend in incidence rates in the experimental group compared to that in the control group, though the effect lacked statistical significance (RR = 0.52, 95% CI 0.23–1.14; p = 0.10), as illustrated in Figure 16.

### 3.7 Drug-related adverse events

**FIGURE 17:**
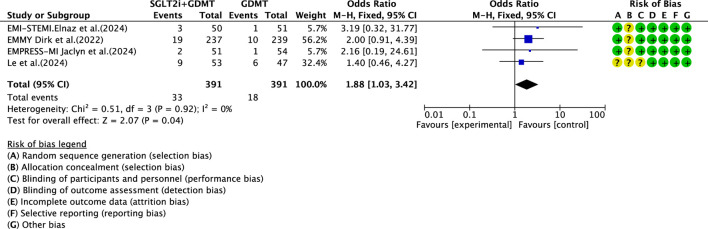
The outcomes of drug adverse reactions of SGLT2i + GDMT vs. GDMT.

Four studies (Zhou et al., 2024; von Lewinski et al., 2022; Carberry et al., 2025; Khani et al., 2024) reported data on drug-related adverse reactions, primarily describing genitourinary infections and hepatic/renal injury, involving 782 patients. Among these, 391 patients formed the experimental group receiving SGLT2i combined with GDMT, while 391 patients formed the control group receiving GDMT alone. The I^2^ test yielded χ^2^ = 0.51, df = 3, p = 0.92, and I^2^ = 0%, enabling meta-analysis using a fixed-effects model. The results indicated a statistically significantly higher rate of incidence in the experimental group compared to that in the control group (RR = 1.88, 95% CI 1.03–3.42; p = 0.04), as illustrated in Figure 17.

### 3.8 Additional subgroup analyses

We conducted subgroup analyses based on the LVEF at admission and the infarct location in patients with AMI to examine the primary outcomes of ST-segment resolution, LVEF, and cTnI (Khani et al., 2024). Specifically, analyses were conducted for baseline LVEF ≤40% *versus* >40% and anterior myocardial infarction (AMI) *versus* non-anterior AMI. Detailed results are presented in Tables 2, 3. As demonstrated in Table 2, patients with AMI in the baseline LVEF ≤40% cohort exhibited markedly greater LVEF improvement following SGLT2i + GDMT treatment compared to the LVEF >40% cohort. In the LVEF >40% group, cTnI levels exhibited a downward trend as early as 8 h post-onset. By 40 h post-onset, cTnI levels showed significant improvement in this group. By 56 h post-onset, cTnI levels also demonstrated significant improvement in the LVEF ≤40% group. As demonstrated in Table 3, LVEF significantly improved in AMI patients treated with SGLT2i + GDMT, irrespective of whether the infarction was anterior or non-anterior. cTnI levels in both groups exhibited a downward trend as early as 16 h post-onset, with non-anterior MI patients demonstrating significant improvement by 24 h post-onset. ST-segment resolution showed no significant difference between the two groups. These findings indicate that SGLT2i combined with GDMT holds potential benefits for early treatment in improving MI.

**TABLE 2:** Subgroups based on LVEF at presentation.

Outcomes	Types of Invention	Subgroup	Heterogeneity				Overall effect						Statistical
			MD	95%CI	z	P	X	df	12(a)	MD	95%CI	P	Method
ST-segment resolution	SGLT2i + GDMT	Baseline LVEF <40%	0.00	[-39.42,39.42]	0.00	1.00	84.68	21.00	75.00	−1.39	[-3.47,0.69]	0.19	Random
		Baseline LVEF >40%	0.00	−19.14.19.15]	0.00	1.00							
LVEF	SGLT21 -GDMT	Baseline LVEF <40%	5.20	[2.74,7.66]	4.15	<0.0001							
		Baseline LVEF >40%	1.80	[-1.43,5.03]	1.09	0.27							
cTnl	5GLT21 -GDMT	Bh, Baseline LVEF <40%	4.10	[-1.05.9.25]	1.56	0.12							
		Bh, Baseline LVEF >40%	0.60	−7.10,8.30]	0.15	0.88							
		16 h, Baseline LVEF <40%	−2.20	[-7.09,2.69]	0.88	0.38							
		16 h Baseline LVEF >40%	−0.40	[-7.36.6.56]	0.11	0.91							
		24 h, Baseline LVEF <40%	−3.70	[-8.04,0.64]	1.67	0.09							
		24 h, Baseline LVEF >40%	−3.40	[-10.63,3.83]	0.92	0.36							
		32 h, Baseline LVEF <40%	0.10	[-3.98,4.18]	0.05	0.96							
		32 h, Baseline LVEF >40%	−2.00	[-7.89.3.89]	0.67	0.51							
		40 h, Baseline LVEF <40%	2.10	−1.50,5.70]	1.14	0.25							
		40 h, Baseline LVEF >40%	−6.10	−11.16,-1.04]	2.36	0.02							
		48 h, Baseline LVEF <40%	−2.20	[-6.31.1.91]	1.05	0.29							
		48 h, Baseline LVEF >40%	−4.50	[-11.24,2.24]	1.31	0.19							
		56 h, Baseline LVEF <40%	−0.40	−3.69,2.89]	0.24	0.81							
		56 h, Baseline LVEF >40%	−8.40	(-13.74.-3.06)	3.08	0.002							
		64 h, Baseline LVEF <40%	3.80	[0.42.7.18]	2.20	0.03							
		64 h, Baseline LVEF >40%	−14.70	−19.60,-9.80	5.88	<0.00001							
		72 h, Baseline LVEF E 40%	−0.50	−4.17,3.17]	0.27	0.79							
		72 h, Baseline LVEF >40%	−2.10	[-10.14.5.94]	0.51	0.61							

**TABLE 3:** Subgroups based on infarct location.

Outcomes	Types of Invention	Subgroup	MD	Heterogeneity			Overall effect						Statistical
				95%CI	z	p	x	df	12(%)	MD	95%CI	p	Method
ST- segment resolution	SGLT2i + GDMT	Anterior MI	2.00	[-15.63,19.63]	0.22	0.82	59.17	21.00	65.00	−1.07	[-2.74,0.60]	<00,001	Random
		Non-anterior MI	−2.00	[-40.70,36.70]	0.10	0.92							
LVEF	SGLT2i + GDMT	Anterior MI	4.20	[0.88,7.52]	2.48	0.01							
		Non-anterior MI	3.90	[0.63,7.17]	2.34	0.02							
cTnI	SGLT2i + GDMT	8 h, Anterior MI	2.00	[-3.48,7.48]	0.72	0.47							
		8 h, Non-anterior MI	6.40	[-0.59,13.39]	1.79	0.07							
		16 h, Anterior MI	−2.80	[-8.00,2.40]	1.05	0.29							
		16 h, Non-anterior MI	−0.10	[-6.41,6.21]	0.03	0.98							
		24 h, Anterior MI	−1.10	[-6.38,4.18]	0.41	0.68							
		24 h, Non-anterior MI	−7.10	[-11.92,-2.28]	2.89	0.004							
		32 h, Anterior MI	−1.60	[-6.58,3.38]	0.63	0.53							
		32 h, Non-anterior MI	0.00	[-3.79,3.79]	0.00	1.00							
		40 h, Anterior MI	0.50	[-4.08,5.08]	0.21	0.83							
		40 h, Non-anterior MI	−3.90	[-6.29,-1.51]	3.20	0.001							
		48 h, Anterior MI	−2.40	[-6.96,2.16]	1.03	0.30							
		48 h, Non-anterior MI	−3.20	[-8.85,2.45]	1.11	0.27							
		56 h, Anterior MI	0.40	[-3.22,4.02]	0.22	0.83							
		56 h, Non-anterior MI	−6.90	[-11.34,-2.46]	3.05	0.002							
		64 h, Anterior MI	0.50	[-4.13,5.13]	0.21	0.83							
		64 h, Non-anterior MI	−6.30	[-9.37,-3.23]	4.03	<0.0001							
		72 h, Anterior MI	1.50	[-2.83,5.83]	0.68	0.50							

### 3.9 Sensitivity analysis

To assess the sensitivity of this meta-analysis, we altered the effect models and observed changes in the MD and SMD under different models. The SMD for LVEF exhibited significant fluctuation, indicating that the results for this indicator may carry a certain degree of risk. The MD (SMD) for other indicators showed only minor fluctuations, suggesting that these results are relatively stable and reliable. A summary of the comparative results is presented in Table 4.

**TABLE 4:** Sensitivity analysis.

SGLT2i + Guideline-directed medical treatment VS. GDMT					
Outcomes	Fixed-effect model	Random-effect model	Outcomes	Fixed-effect model	Random-effect model
LVEF	0.12[0.04,0.21]	0.65[0.17,1,13]	LVEDV	−0.04[-0.21,0.12]	−0.04[-0.21,0.12]
NT-ProBNP	−0.09[-0.20,0.03]	−0.93[-1.72,-0.14]	LAV	−0.19[-0.31,-0.07]	−0.19[-0.31,-0.07]
LVESVI	0.04[-0.08,0.15]	0.04[-0.08,0.15]	LV mass index	−0.22[-0.45,0.00]	−0.22[-0.57,0.12]
LVEDVI	0.07[-0.05,0.18]	0.07[-0.05,0.18]	LVM	0.09[-0.18,0.36]	0.09[-0.18,0.36]
LAVI	−0.17[-0.29,-0.04]	−0.17[-0.29,-0.04]	LVMI	0.22[-0.05,0.49]	0.22[-0.05,0.49]
LVESD	−0.05[-0.26,0.15]	−0.07[-0.83,0.68]	ePASP	0.39[0.16,0.62]	0.41[-0.22,1.04]
LVEDD	0.04[-0.07,0.16]	0.16[-0.44,0.75]	cTnl	−0.11[-0.24,0.01]	−0.11[-0.24,0.01]
LVESV	−0.06[-0.18,0.06]	−0.06[-0.18,0.06]	hs-CRP	−0.02[-0.30,0.26]	−0.02[-0.30,0.26]
MacNew HRQoL	0.19[-0.00,0.39]	0.19[-0.00,0.39]			

### 3.10 Meta regression

Given the substantial heterogeneity observed for LVEF (I^2^ = 99%), LVESD (I^2^ = 92%), and LVEDD (I^2^ = 94%), meta-regression analyses were conducted to further investigate potential sources of variability across studies. Study-level covariates, including follow-up duration and baseline at admission, were entered into the regression model using a random-effects framework (REML method). The analysis revealed that the baseline LVEF and follow-up duration were not significant moderators (all p > 0.05). These findings indicate that the observed heterogeneity primarily originates from differences in the study design and clinical settings rather than from inconsistencies in the treatment effect itself. When combined with the results of the sensitivity and subgroup analyses, the meta-regression supports the overall robustness and internal consistency of the pooled estimates. Detailed results are provided in Supplementary Table S3.

### 3.11 Publication bias analysis

Publication bias was assessed using the Egger test and Harbord method, and the results are presented in Table 5. We focused on relatively important outcome measures. Publication bias for LVEF was evaluated using the Egger test. The results showed t = 0.51, 95% CI −44.95312 to 48.75085, and p = 0.697. For NT-proBNP levels, the Egger test yielded t = −1.55, 95% CI −106.8187 to 83.53777, and p = 0.364. Both results indicated no publication bias, lending credibility to the findings. Additionally, the Harbord’s test was employed to assess the publication bias for MACE, re-admission for HF, and drug-related adverse events. The Harbord’s test for MACE yielded t = 1.53, with a 95% CI of −67.59383 to 86.11274 and p = 0.368. For re-admission for HF, the Harbord’s test yielded t = 1.96, 95% CI −2.253835 to 6.017914, and p = 0.189. For drug-related adverse reactions, the Harbord’s test yielded t = 0.76, with a 95% CI of −3.739081 to 5.341258 and p = 0.527. These results indicate no publication bias, thus lending credibility to the conclusions. Due to insufficient detail in data such as LVEDD, LVESD, LVESV, LVESVI, LVEDV, and LVEDVI, no publication bias analysis was conducted.

**TABLE 5:** Publication bias.

Egger’s tests(P)		Harbord’s tests(P)		
LVEF	NT-ProBNP	MACE	Re-admission for heart failure	Adverse drug reaction
t = 0.51 95% CI -44.95312 to 48.75085	t = −1.55 95% CI -106.8187 to 83.53777	t = 1.53 95% CI -67.59383 to 86.11274	t = 1.96 95% CI -2.253835 to 6.017914	t = 0.76 95% CI -3.739081 to 5.341258
p = 0.697	p = 0.364	p = 0.368	p = 0.189	p = 0.527

### 3.12 Assessment of evidence quality

**FIGURE 18:**
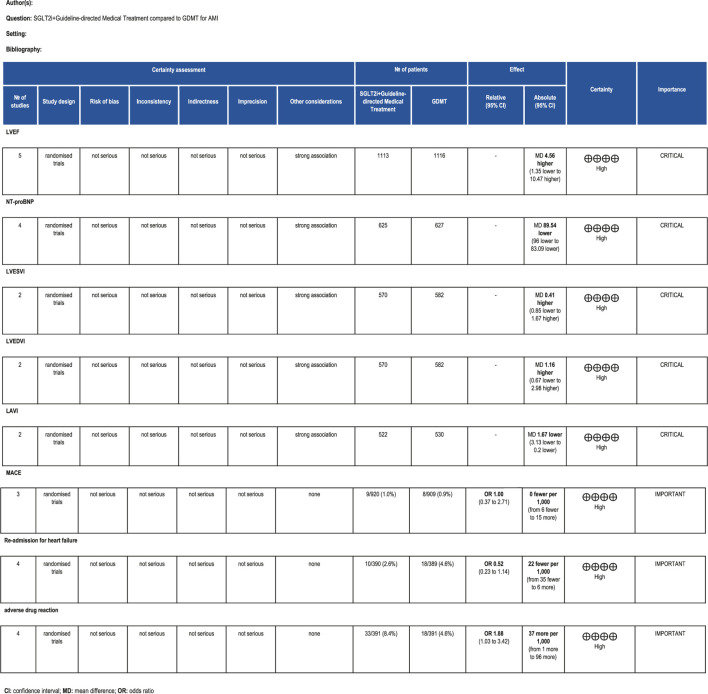
The Gradeprofile of SGLT2i + GDMT vs. GDMT for main outcomes.

The GRADE framework was employed to evaluate the quality of evidence for primary outcome measures. Findings are summarized as follows: For LVEF scores, the certainty of evidence was rated as high. For NT-proBNP, the certainty of evidence was rated as high. For LVESVI, the certainty of evidence was rated as high. For LVEDVI, the certainty of evidence was rated as high. LAVI had high certainty of evidence. MACE had high certainty of evidence. Re-admission for HF had high certainty of evidence. Drug-related adverse events had high certainty of evidence. The relevant assessment results are presented in Figure 18.

## 4 Discussion

### 4.1 Summary of the primary outcome measures

This study ultimately included five RCTs meeting the inclusion criteria, involving a total of 881 participants. All results are summarized in Table 6. The primary outcome measures assessed were LVEF, NT-proBNP, LVESVI, LVEDVI, and LAVI—key indicators of ventricular remodeling prognosis in AMI patients. Among the five RCTs evaluating LVEF, the SGLT2i + GDMT group showed an upward trend compared to the control group. An additional investigation was conducted in which LVEF was subdivided according to the stages of treatment. In the 4-week subgroup, the experimental group exhibited an upward trend in LVEF, with a significant increase at 12 weeks compared to that in the control group (RR = 6.32, 95% CI −4.95 to 17.60; p < 0.00001). By 26 weeks, the two groups were largely comparable, suggesting that SGLT2i may aid in restoring cardiac pumping function. In the four RCTs evaluating NT-proBNP, the SGLT2i + GDMT group demonstrated a significant reduction compared to the control group. Subgroup analysis was conducted based on the treatment duration. In the 12-week subgroup, NT-proBNP levels decreased significantly in the experimental group (RR = −89.82, 95% CI −96.28 to −83.35; p < 0.00001). In the 26-week subgroup, NT-proBNP showed a decreasing trend in the experimental group. This indicates that SGLT2 inhibitors have a beneficial effect in reducing NT-proBNP (a cardiac stress marker). This outcome exhibits the same trend as LVEF. In the two RCTs evaluating LVESVI and LVEDVI, the SGLT2i + GDMT group showed a decreasing trend compared to the control group. In the two RCTs evaluating LAVI, the SGLT2i + GDMT group showed a significant reduction compared to the control group (RR = −1.67, 95% CI −3.13 to −0.20; p = 0.03). The two studies were subdivided into two subgroups based on the treatment process. In the 6-week subgroup, LAVI showed a trend toward reduction in the experimental group; while in the 26-week subgroup, LAVI was significantly reduced in the experimental group. Previous studies only assessed the benefits of SGLT2i on cardiac function at the conclusion of treatment, failing to examine specific changes in cardiac function throughout the recovery period. Based on these findings, it is inferred that early application of SGLT2i during the acute phase of AMI may aid in the recovery of cardiac function and structure.

**TABLE 6:** Total outcomes and primary end points.

Outcomes	Types of Intervention	Subgroup	Heterogeneity				Overall effect				Statistical method	Studies(N)	Participants(N)	Figures
			MD	95%CI	z	p	x	df	12(%)	p				
Primary outcomes														
LVEF	SGLT2i + GDMT	total	4.56	[-1.35,10.47]	1.51	0.13	1077.3	10	99	0.13	Random	5	881	S5
		Baseline	8.68	[-7.56,24.92]	1.05	0.29								
		4 weeks	1.44	[-0.90,3.77]	1.2	0.23								
		12 weeks	6.32	[-4.95,17.60]	1.1	0.27								
		26 weeks	−1.80	[-1.76,1.40]	0.22	0.82								
NT-proBNP	SGLT2i + GDMT	total	−89.54	[-96.00,-83.09]	27.19	<0.00001	4.21	4	5	<0.00001	Fixed	4	780	S6
		12 weeks	−89.82	[-96.28,-83.35]	27.23	<0.00001								
		26 weeks	−1.70	[-117.30,113.90	0.03	0.98								
LVESVI	SGLT2i + GDMT	total	0.41	[-0.85,1.67]	0.64	0.52	2.98	3	0	0.52	Fixed	2	580	S7
		Baseline	1.12	[-0.49,2.73]	1.36	0.17								
		26 weeks	−0.72	[-2.75,1.32]	0.69	0.49								
LVEDVI	SGLT2i + GDMT	total	1.16	[-0.67,2.98]	1.24	0.21	1.04	3	0	0.21	Fixed	2	580	S8
		Baseline	1.82	[-0.52,4.17]	1.52	0.13								
		26 weeks	0.14	[-2.77,3.05]	0.09	0.92								
LAVI	SGLT2i + GDMT	total	−1.67	[-3.13,-0.20]	2.23	0.03	3.21	2	38	0.03	Fixed	2	580	S9
		6 weeks	−1.05	[-2.73,0.63]	1.23	0.22								
		26 weeks	−3.66	[-6.66,-0.65]	2.39	0.32								
Secondary outcomes														
LVESD	SGLT2i + GDMT	total	−0.68	[-1.58,0.21]	1.49	0.14	39.54	3	92	0.14	Random	2	200	S10
		Baseline	−0.14	[-1.20,0.92]	0.26	0.8								
		12 weeks	−2.19	[-7.55,3.17]	0.8	0.42								
LVEDD	SGLT2i + GDMT	total	−0.57	[-1.68,0.53]	1.02	0.31	71.34	4	94	0.31	Random	3	676	S11
		4 weeks	0.50	[-0.14,1.13]	1.52	0.13								
		12 weeks	−2.88	[-10.84,5.08]	0.71	0.48								
		26 weeks	−0.29	[-1.23,0.65]	0.61	0.54								
LVESV	SGLT2i + GDMT	total	−1.54	[-4.22,1.15]	1.12	0.26	0.66	2	0	0.26	Fixed	2	580	S12
		4 weeks	−1.41	[-5.35,2.53]	0.7	0.48								
		26 weeks	−1.54	[-5.30,2.01]	0.88	0.38								
LVEDV	SGLT2i + GDMT	total	−1.79	[-7.89,4.32]	0.57	0.57	0.13	1	0	0.57	Fixed	2	580	S13
LAV	SGLT2i + GDMT	total	−3.86	[-6.33,-1.38]	3.06	0.002	0.41	2	0	0.002	Fixed	2	580	S14
		6 weeks	−3.10	[-6.59,0.39]	1.74	0.08								
		26 weeks	−4.61	[-8.11,-1.12]	2.59	0.01								
Primary endpoints														
MACE	SGLT2i + GDMT	total	1.00	[0.37,2.71]	0.00	1.00	3.84	5	0	1.00	Fixed	3	677	S15
Re-admission for heart failure	SGLT2i + GDMT	total	0.52	[0.23,1.14]	1.64	0.10	1.79	3	0	0.10	Fixed	4	779	S16
Drug-related adverse events	SGLT2i + GDMT	total	1.88	[1.03,3.42]	2.07	0.04	0.51	3	0	0.04	Fixed	4	782	S17

Regarding the secondary endpoint of LAV, data from two trials demonstrated that the SGLT2i + GDMT group exhibited a significant reduction compared to the control group (RR = −3.86, 95% CI −6.33 to −1.38; p = 0.002). The two studies were subdivided into two subgroups based on the treatment pathways. In the 6-week subgroup, the SGLT2i + GDMT group showed a trend toward reduced LAV compared with the control group. In the 26-week subgroup, the SGLT2i + GDMT group demonstrated a significant reduction in LAV compared with the control group (RR = −4.61, 95% CI −8.11 to −1.12; p = 0.010). In the three RCTs assessing LVEDD, the SGLT2i + GDMT group showed a trend toward reduction compared with the control group. Two studies were subdivided into subgroups based on the treatment duration. A trend toward a reduction in LVEDD was observed in the experimental group at both 4 and 12 weeks. Data from two trials assessing LVESD, LVESV, and LVEDV indicated a trend toward reduction in the SGLT2i + GDMT group compared with the control group.

The improvements in NT-proBNP and LAVI observed in this analysis likely reflect the integrated cardioprotective mechanisms of SGLT2is beyond glucose lowering. Current experimental and clinical evidence suggests that these agents modulate cardiac remodeling through multiple pathways. At the neuro-hormonal level, SGLT2 inhibition attenuates sympathetic overactivity. Coupled with its cardiorenal benefits, this leads to an overall reduction in ventricular wall stress and neurohormonal burden after MI, despite potential transient and modest effects on the RAAS. At the cellular level, SGLT2is improve myocardial energetics by enhancing ketone body utilization and promoting mitochondrial efficiency, which supports ATP production and reduces oxidative injury during ischemia–reperfusion stress. In parallel, suppression of the sodium–hydrogen exchanger-1 (NHE-1) and downstream calcium overload may stabilize cardiomyocyte electrophysiological homeostasis. Furthermore, accumulating evidence indicates that SGLT2 inhibition mitigates inflammation, oxidative stress, and ferroptosis, all of which contribute to maladaptive post-infarction remodeling. Collectively, these mechanisms converge to preserve the myocardial structure and function, providing a plausible biological basis for the observed reductions in NT-proBNP and LAVI and the sustained improvement in LVEF. These integrated effects may explain the consistent improvements observed across functional and biomarker endpoints.

The primary endpoint assessed in the study was MACE—a key indicator of prognosis in patients with AMI. The incidence rates in the SGLT2i + GDMT group were broadly comparable to those in the control group. Analysis of three MACE subgroups showed the following: cardiovascular disease-related death (RR = 1.47, 95% CI 0.29–7.56; p = 0.64), recurrent MI (RR = 0.64, 95% CI 0.16–2.55; p = 0.53), and stroke (RR = 2.71, 95% CI 0.11–68.25; p = 0.54). When evaluating the primary outcome of re-admission for HF, the incidence rate in the experimental group showed a downward trend compared to the control group. Regarding drug-related adverse events, the experimental group exhibited a statistically significantly higher incidence rate than the control group (RR = 1.88, 95% CI 1.03–3.42; p = 0.04). This indicates that SGLT2is may confer cardiac benefits in AMI patients, though drug-related adverse reactions warrant continued attention. Although the study did not report significant hepatic or renal dysfunction, there was an increased incidence of genitourinary infections and acute kidney injury. Most of these adverse reactions are preventable and manageable. In clinical practice, attention should be paid to the patient’s baseline condition (e.g., renal function and blood volume), monitoring for signs of infection, renal function, and blood glucose during treatment and being vigilant regarding the risk of hypoglycemia when co-administering other medications.

Results from other subgroup analyses are as follows: Subgroup analyses were conducted for primary outcomes (ST-segment resolution, LVEF, and cTnI) based on the LVEF at admission and the infarct location in patients with AMI. Regarding the magnitude of LVEF improvement, patients with AMI and an admission LVEF ≤40% demonstrated markedly greater LVEF improvement in the SGLT2i + GDMT treatment group (MD = 5.20, 95% CI 2.74 to 7.66; p < 0.0001) compared to those with LVEF >40%. In the group with LVEF >40% at admission, cTnI levels exhibited a downward trend as early as 8 h post-onset. By 40 h post-onset, cTnI levels showed significant improvement in this group (MD = −6.10, 95% CI −11.16 to −1.04; p = 0.02). Significant improvement in cTnI levels was observed in the LVEF ≤40% group after 56 h post-onset (MD = −8.40, 95% CI −13.74 to −3.06; p = 0.002). Regarding the impact of the infarct location on LVEF recovery, whether anterior wall (MD = 4.20, 95% CI 0.88 to 7.52; p = 0.01) or non-anterior wall MI (MD = 3.90, 95% CI 0.63 to 7.17; p = 0.02), LVEF showed significant improvement in AMI patients treated with SGLT2i + GDMT. cTnI levels exhibited a downward trend in both groups as early as 16 h post-onset. In non-anterior MI patients, cTnI levels showed significant improvement by 24 h post-onset (MD = −1.70, 95% CI −11.92 to −2.28; p = 0.004). However, no significant difference in ST-segment resolution was observed between the two groups. These findings suggest that SGLT2i combined with GDMT may offer potential benefits in terms of improving heart muscle function in patients with early-stage HF, particularly among those with more extensive heart muscle damage.

Additionally, multiple trials assessed several secondary outcome measures. Regarding the LV mass index, the SGLT2i + GDMT group showed a trend toward reduction at 4 weeks compared with the control group, with a significant reduction observed at 12 weeks (RR = −9.93, 95% CI −17.58 to −2.28; p = 0.01). Regarding cTnI, the SGLT2i + GDMT group showed a significant reduction compared with that in the control group at 12 weeks (RR = −1.21, 95% CI −2.34 to −0.08; p = 0.04). Subgroup analysis further confirmed that the SGLT2i + GDMT group showed a decreasing trend compared with the control group at 16 h post-onset, with a significant reduction at 24 h (RR = −3.40, 95% CI −7.14 to 0.34; p = 0.07). No statistically significant differences were observed between the two groups in LVM, LVMI, ePASP, or hs-CRP before and after treatment. Regarding MacNew HRQoL scores, the SGLT2i + GDMT group demonstrated significant improvement compared with the control group (RR = 0.16, 95% CI 0.01–0.31; p = 0.04). This indicates that SGLT2i improves the quality of life and functional status in AMI patients, likely by alleviating the physical, emotional, and social stressors.

Sensitivity analysis indicates that primary outcome measures largely align with baseline findings, confirming that SGLT2is may effectively mitigate cardiac remodeling following AMI and associated adverse cardiovascular events. Publication bias analysis reveals no evidence of publication bias, lending credibility to the conclusions.

### 4.2 Sources of heterogeneity

Sources of heterogeneity in this study included the following: 1) Two studies employed block randomization, two utilized computer-generated randomization methods, one did not specify the randomization method, and the remaining RCTs did not mention allocation concealment. 2) Four studies employed double-blind designs, while the remaining RCTs did not mention blinding. 3) Two RCTs had incomplete outcome data, all constituting sources of publication bias. 4) Heterogeneity was observed in LVEF outcomes. Subgroup analysis by reperfusion time revealed significant heterogeneity in the 4-week and 26-week subgroups; differences in the treatment duration may be a source of heterogeneity. 5) Heterogeneity was observed in LVESVI and LVEDVI outcomes. Subgroup analysis by reperfusion time still revealed heterogeneity, which is potentially attributable to differing treatment cycles, operating equipment, and diagnostic criteria. 6) Heterogeneity existed in LVM, LVMI, ePASP, and hs-CRP levels, likely stemming from variations in treatment protocols, diagnostic standards, testing reagents, and laboratory conditions. 7) Sensitivity analysis revealed considerable fluctuations in the SMD for LVEF, indicating potential risk. 8) Ultrasound assessment carries inherent subjectivity, with potential implementation and measurement biases during result interpretation. 9)Although a high degree of heterogeneity (I^2^ = 99%) was identified in the pooled LVEF、LVESD、LVEDD analysis, further subgroup, sensitivity, and meta-regression analyses confirmed that the direction of benefit remained consistent across studies. The subgroup analysis showed that improvements in LVEF were observed regardless of baseline LVEF and infarct location, though the magnitude of improvement varied slightly. The sensitivity analysis demonstrated that no single trial significantly altered the overall results, confirming that the findings were not driven by an individual study. Importantly, meta-regression revealed that longer follow-up duration was modestly associated with greater recovery in LVEF, whereas baseline cardiac function and follow-up duration had no significant influence.

Taken together, these analyses strengthen the reliability of our findings and support the hypothesis that SGLT2is exert a consistent cardioprotective effect following MI. Nevertheless, future large-scale RCTs with longer follow-up are necessary to validate these observations and clarify long-term outcomes.

### 4.3 Safety of SGLT2 inhibitors

Meta-analysis of safety data from the included trials indicated that SGLT2is were associated with a significantly increased risk of drug-related adverse events (RR = 1.88, 95% CI 1.03–3.42; p = 0.04). This risk was primarily driven by a higher incidence of genitourinary infections, which is consistent with the established pharmacologic profile of this drug class. The mechanism is attributed to glucosuria, which creates a favorable environment for microbial growth in the genital and urinary tracts. Notably, these infections were generally mild to moderate in severity, responsive to standard antimicrobial therapy, and rarely led to treatment discontinuation in clinical trials. Preventive measures, such as maintaining personal hygiene, are recommended, especially for patients with a history of recurrent infections.

Regarding renal effects, initiation of SGLT2i therapy is frequently associated with a transient, reversible decline in estimated glomerular filtration rate (eGFR) and a concomitant small increase in serum creatinine. This is largely considered a hemodynamic adaptation (reduced glomerular hyperfiltration) rather than a marker of intrinsic renal injury. Importantly, large-scale cardiovascular and HF outcome trials have consistently demonstrated that SGLT2is confer significant long-term renal protective benefits, slowing the progression of chronic kidney disease. In the context of post-MI, trials such as EMMY and EMPRESS-MI have not reported an excess of serious renal adverse events, supporting their acceptable renal safety profile with appropriate monitoring (e.g., assessing renal function at initiation and periodically thereafter). No consistent signal of drug-induced liver injury has been observed across major clinical trial programs.

In conclusion, the safety profile of SGLT2is in post-MI patients appears to be manageable and consistent with their known effects in other populations. The documented cardiorenal benefits outweigh the risks of generally manageable side effects such as genitourinary infections. Clinicians should incorporate preventive strategies and early monitoring to mitigate these risks while maximizing the therapeutic potential of this drug class.

### 4.4 Strengths and limitations of the study

This study represents the most recent meta-analysis to date evaluating the efficacy and safety of SGLT2is combined with GDMT, providing further evidence for the clinical application of SGLT2is as adjunctive therapy in patients with AMI. Subgroup analyses based on the treatment duration were conducted for AMI patients, alongside sensitivity analyses and publication bias assessments. These findings are consistent with the broader landscape of evidence for SGLT2is. While large HF trials (e.g., EMPA-REG OUTCOME and DAPA-HF) established their efficacy in reducing hard clinical endpoints, our results provide mechanistic insights by demonstrating early improvements in cardiac remodeling parameters in post-MI patients. These improvements in LVEF, NT-proBNP, and cardiac structure are the putative precursors to the long-term clinical benefits. Furthermore, our results directly align with the findings of recent dedicated post-MI trials such as EMMY and EMPRESS-MI (Carberry et al., 2025; von Lewinski et al., 2022), reinforcing the role of SGLT2is in attenuating adverse ventricular remodeling following an acute infarction.

This study represents the most recent meta-analysis to date evaluating the efficacy and safety of SGLT2is combined with GDMT, providing further evidence for the clinical application of SGLT2is as adjunctive therapy in patients with AMI. Subgroup analyses based on treatment duration were conducted for AMI patients, alongside sensitivity analyses and publication bias assessments. However, several limitations should be acknowledged. First, the current number of RCTs is limited, with a total of only five studies and 881 patients included. This restricts the statistical power for some secondary endpoints, particularly for assessing rare adverse events and robust subgroup comparisons. Second, by design, we restricted our inclusion criteria to RCTs to ensure internal validity and robust causal inference. While this strengthens our conclusions regarding efficacy, it may limit the immediate generalizability of our findings to broader, real-world populations that often include patients excluded from RCTs. Furthermore, this focus may underrepresent long-term safety data that are better captured in observational designs. It is encouraging, however, that our findings are complemented by emerging real-world evidence. For instance, the large international SGLT2-I AMI PROTECT registry (Paolisso et al., 2023) similarly identified SGLT2i use as an independent predictor of reduced MACEs, corroborating the direction of benefit observed in our analysis. Third, the included studies had a relatively short follow-up period, with the longest being 26 weeks. This precludes the possibility of definitive conclusions about the long-term sustainability of the observed benefits on cardiac remodeling and requires further investigation into whether these early structural and biomarker improvements translate into long-term reductions in hard clinical endpoints. Fourth, significant heterogeneity was observed in certain results (e.g., LVEF), which is primarily attributable to variations in follow-up duration, treatment protocols, and measurement techniques. Although we employed random-effects models and conducted subgroup analyses to address this, residual heterogeneity remains a consideration. Fifth, the methodological quality of some included trials presented unclear risks of bias concerning random sequence generation, allocation concealment, and blinding implementation, which may potentially influence the results. Finally, although subgroup analyses indicated greater benefit in the early phase of AMI and among patients with severe baseline cardiac impairment, the precise optimal timing of initiation, ideal treatment duration, and the dominant mechanisms of benefit in this specific population remain unclear. In conclusion, while the current evidence base suggests that SGLT2i intervention may ameliorate early ventricular remodeling, the findings should be interpreted with caution due to the limited scale and duration of the existing studies. Future large-scale, long-term, and rigorously designed RCTs are urgently needed to confirm these findings, define optimal treatment strategies, and verify the translation of these benefits into improved long-term clinical outcomes.

## 5 Conclusion

Available data suggest that SGLT2is are associated with improvements in LVEF, reductions in ventricular volumes, and favorable changes in NT-proBNP among patients following MI. These findings suggest a potential attenuation of early ventricular remodeling, which is consistent with the mechanistic benefits observed in recent randomized trials.

## Data Availability

The original contributions presented in the study are included in the article/Supplementary Material, further inquiries can be directed to the corresponding author.
