# Supplementary material for: The impact of SGLT2 inhibitors on cardiac remodeling after myocardial infarction: an updated meta-analysis of randomized controlled trials
Source: Front Pharmacol. 2025 Nov 27;16:1699066. doi: 10.3389/fphar.2025.1699066 (PMC12696861; doi:10.3389/fphar.2025.1699066)
Supplement: Supplementary file 1 [file Supplementaryfile1.zip › Data Sheet2/Supplementary Table S1.docx]

**Table S1.** Search Strategies

The following databases were searched from their inception to May 2025:

| **PubMed** | 1.(sglt2 inhibitors or canagliflozin or dapagliflozin or empagliflozin) AND myocardial infarction  2.((sglt2 inhibitors or canagliflozin or dapagliflozin or empagliflozin) AND myocardial infarction) OR (Post-MI)  3.([Sodium-Glucose Transporter 2 Inhibitors](https://research.ebsco.com/c/bclpce/search/results?db=mdl&isDashboardExpanded=true&limiters=None&q=MM+%22Sodium-Glucose+Transporter+2+Inhibitors%22&redirectFromDetailsToResultsPage=true&initiatedBy=typed-in) or canagliflozin or dapagliflozin or empagliflozin) AND myocardial infarction  4.(([Sodium-Glucose Transporter 2 Inhibitors](https://research.ebsco.com/c/bclpce/search/results?db=mdl&isDashboardExpanded=true&limiters=None&q=MM+%22Sodium-Glucose+Transporter+2+Inhibitors%22&redirectFromDetailsToResultsPage=true&initiatedBy=typed-in) or canagliflozin or dapagliflozin or empagliflozin) AND myocardial infarction) OR (Post-MI)  5.(sglt2 inhibitors or canagliflozin or dapagliflozin or empagliflozin) AND myocardial infarction OR(cardiac remodeling) |
| --- | --- |
| **Cochrane library** | 1 [Sodium-Glucose Transporter 2 Inhibitors](https://research.ebsco.com/c/bclpce/search/results?db=mdl&isDashboardExpanded=true&limiters=None&q=MM+%22Sodium-Glucose+Transporter+2+Inhibitors%22&redirectFromDetailsToResultsPage=true&initiatedBy=typed-in)  2 sglt2 inhibitors  3 canagliflozin or dapagliflozin or empagliflozin or Ertugliflozin，Bexagliflozin，Luseogliflozin，Tofogliflozin，Ipragliflozin**，**sotagliflozin  4 1 or 2 or 3  5 acute myocardial infarctions  6 acute myocardial infarction  7 myocardial infarction  8 AMI  9 cardiac remodeling  10 5 or 6 or 7 or 8 or 9  11 randomized controlled trial  12 single blind procedure or double blind procedure  13 10 or 11  14 4 and 10 and 13 |
| **EMBASE** | 1 ' [Sodium-Glucose Transporter 2 Inhibitors](https://research.ebsco.com/c/bclpce/search/results?db=mdl&isDashboardExpanded=true&limiters=None&q=MM+%22Sodium-Glucose+Transporter+2+Inhibitors%22&redirectFromDetailsToResultsPage=true&initiatedBy=typed-in) '  2 ' glt2 inhibitors'  3 (' canagliflozin ' or ' dapagliflozin ' or ' empagliflozin ' or ' Ertugliflozin ' or ' Bexagliflozin ' or ' Luseogliflozin ' or ' Tofogliflozin ' or ' Ipragliflozi').ti,ab.  4 1 or 2 or 3  5 ' acute myocardial infarctions '  6 ' acute myocardial infarction '  7 ' myocardial infarction '  8 'AMI '  9 cardiac remodeling  10 5 or 6 or 7 or 8 or 9  11 randomized controlled trial  12 single blind procedure or double blind procedure  13 10 or 11  14 4 and 10 and 13 |
| **Web of Science** | (sodium-Glucose Transporter 2 Inhibitors OR sglt2 inhibitors OR canagliflozin OR dapagliflozin OR empagliflozin OR Ertugliflozin OR Bexagliflozin OR Luseogliflozin OR Tofogliflozin OR Ipragliflozin OR sotagliflozin)  AND  (acute myocardial infarctions OR acute myocardial infarction OR myocardial infarction OR AMI OR cardiac remodeling)  AND  (randomized controlled trial [pt] OR controlled clinical trial [pt] OR trial [tiab] OR clinical trials as topic [mesh: noexp] OR Clinical Trial OR random* [tiab] OR random allocation [mh] OR single-blind method [mh] OR double-blind method [mh]) |
| **Medline** | (sodium-Glucose Transporter 2 Inhibitors OR sglt2 inhibitors OR canagliflozin OR dapagliflozin OR empagliflozin OR Ertugliflozin OR Bexagliflozin OR Luseogliflozin OR Tofogliflozin OR Ipragliflozin OR sotagliflozin)  AND  (acute myocardial infarctions OR acute myocardial infarction OR myocardial infarction OR AMI OR cardiac remodeling)  AND  (randomized controlled trial OR controlled clinical trial OR trial OR Clinical Trial OR random* OR random allocation OR single-blind method OR double-blind method) |
| **Clinicaltrials.gov** | Condition or disease: acute myocardial infarctions OR acute myocardial infarction OR myocardial infarction OR AMI  Other terms: (sodium-Glucose Transporter 2 Inhibitors OR sglt2 inhibitors OR canagliflozin OR dapagliflozin OR empagliflozin OR Ertugliflozin OR Bexagliflozin OR Luseogliflozin OR Tofogliflozin OR Ipragliflozin OR sotagliflozin) |
| **the WHO Clinical Trials Registry Platform** | 1 [Sodium-Glucose Transporter 2 Inhibitors](https://research.ebsco.com/c/bclpce/search/results?db=mdl&isDashboardExpanded=true&limiters=None&q=MM+%22Sodium-Glucose+Transporter+2+Inhibitors%22&redirectFromDetailsToResultsPage=true&initiatedBy=typed-in)  2 sglt2 inhibitors  3 canagliflozin or dapagliflozin or empagliflozin or Ertugliflozin，Bexagliflozin，Luseogliflozin，Tofogliflozin，Ipragliflozin**，**sotagliflozin  4 1 or 2 or 3  5 acute myocardial infarctions  6 acute myocardial infarction  7 myocardial infarction  8 AMI  9 cardiac remodeling  10 5 or 6 or 7 or 8 or 9  11 randomized controlled trial  12 single blind procedure or double blind procedure  13 10 or 11  14 4 and 10 and 13 |
